# A novel interpretable machine learning model approach for the prediction of TiO_2_ photocatalytic degradation of air contaminants

**DOI:** 10.1038/s41598-024-62450-z

**Published:** 2024-06-06

**Authors:** Rodrigo Teixeira Schossler, Samuel Ojo, Zhuoying Jiang, Jiajie Hu, Xiong Yu

**Affiliations:** 1 Department of Civil and Environmental Engineering, Case Western Reserve University, Bingham Building-Room 237, Cleveland, OH 44106 USA; 2 Department of Electrical Engineering and Computer Science (courtesy appointment), Case Western Reserve University, Bingham Building-Room 237, Cleveland, OH 44106 USA; 3 Department of Mechanical and Aerospace Engineering (Courtesy Appointment), Case Western Reserve University, Bingham Building-Room 237, Cleveland, OH 44106 USA

**Keywords:** Photo-degradation of air contaminants, Titanium dioxide, Machine learning, Molecular fingerprint, Reaction rate constant, Environmental impact, Chemical engineering

## Abstract

Air contaminants lead to various environmental and health issues. Titanium dioxide (TiO_2_) features the benefits of autogenous photocatalytic degradation of air contaminants. To evaluate its performance, laboratory experiments are commonly used to determine the kinetics of the photocatalytic-degradation rate, which is labor intensive, time-consuming, and costly. In this study, Machine Learning (ML) models were developed to predict the photo-degradation rate constants of air-borne organic contaminants with TiO_2_ nanoparticles and ultraviolet irradiation. The hyperparameters of the ML models were optimized, which included Artificial Neural Network (ANN) with Bayesian optimization, gradient booster regressor (GBR) with Bayesian optimization, Extreme Gradient Boosting (XGBoost) with optimization using Hyperopt, and Catboost combined with Adaboost. The organic contaminant was encoded through Molecular fingerprints (MF). Imputation method was applied to deal with the missing data. A generative ML model Vanilla Gan was utilized to create synthetic data to further augment the size of available dataset and the SHapley Additive exPlanations (SHAP) was employed for ML model interpretability. The results indicated that data imputation allowed for the full utilization of the limited dataset, leading to good machine learning prediction performance and preventing common overfitting problems with small-sized data. Additionally, augmenting experimental data with synthetic data significantly improved prediction accuracy and considerably reduced overfitting issues. The results ranked the feature importance and assessed the impacts of different experimental variables on the rate of photo-degradation, which were consistent with physico-chemical laws.

## Introduction

Increases in global urbanization and industrial growth have led to a rise in the emissions rate of toxic compounds into the air and waterways, which has caused diseases, global warming, and abnormal climatic conditions. Air pollutants in the indoor environment can result in sick home syndrome^1^. In addition, emissions of air pollutants contribute to the formation of environmental hazards like stratospheric ozone depletion, urban smog and ozone, and the greenhouse effect. The threat posed by airborne pollutants on human health and the environment calls for innovative strategies for their mitigation. Titanium dioxide (TiO_2_) for heterogeneous photocatalysis is a renowned technique for the purification of contaminants; its nonselective photocatalytic feature opens it up to a great extent in destroying contaminants with a wide range of functional groups and structures. Previous work has shown promising results of TiO_2_ in the degradation of both waterborne and airborne pollutants^2,3^. Advantages of TiO_2_ include its cost-effectiveness, low energy consumption, high efficiency, featuring good oxidizing power; it is also chemically stable and resistant to acid, easily produced, and not soluble in water. Results have shown it is efficient in the destruction of pollutants; the photocatalytic process can operate at ambient temperature and pressure^1,4,5^. Furthermore, TiO_2_ applications range from the purification of air, treatment of water, water splitting, renewable energy processes, and the conversion of carbon dioxide to hydrocarbon^6–9^.

Photocatalyst efficiency is affected by many factors, including but not limited to pH, photocatalyst dosage, inorganic ions in leachate, light intensity, types of elements used for doping, the content of the dopant, photocatalyst phase, the synthesis temperature of the photocatalyst phase, mixing intensity, and dissolved oxygen^10–13^. Some important experimental features, including the initial concentration of the contaminant, solution’s pH value, TiO_2_ dosage, humidity, and temperature, have been studied to evaluate their impacts on the photo-degradation efficiency^14^. However, the implementation of experiments is expensive and requires dedicated equipment; it is also time-consuming and labor-intensive. Based on the lattice characteristics and surface area, previous research employed the Gaussian process regression model to predict the anatase TiO_2_ photocatalyst band gaps^15^. Such model predictions may not describe in detail the full performance of the TiO_2_ in terms of light harvesting ability, light absorption, and photo-degradation ability, since not all factors affecting photo-degradation are evaluated.

Computational modeling, based on the collection of data from published sources, provides a cost-effective method to determine the effectiveness of photocatalytic degradation of an organic contaminant with UV and TiO_2_ treatment^16^. Various computational techniques, including neural network-based multivariate, the response surface method (RSM) and generic programming (GP), have been applied to predict the rate at which water pollutants degrade^17–21^. RSM faces some limitations, such as the difficulty in estimating the accuracy of an approximation. The developed response is limited to the regions within the studied ranges of factors and is inappropriate for optimizing problems with highly nonlinear multiple objectives. It is only suitable for quadratic and linear approximations. This means that some complex physical phenomena may encounter difficulties because the objective function must be continuously differentiable^22^.

The progress in data science introduces machine learning (ML) models for assessing photocatalyst performance. The application of machine learning allows for the assessment of photocatalytic performance in a way that is faster, cheaper, and more flexible than conventional experimental approaches^23^. ML models can be utilized to reveal hidden patterns among existing data and predict the properties and performance of materials^24^. These include the use of Artificial Neural Network (ANN) as a predictive tool to determine catalyst performance, considering the impacts of various contaminants, properties of materials, and experimental variables^21,25,26^.

In this study, an investigation is conducted on developing interpretable machine learning (ML) to predict the photo-degradation performance of TiO_2_ on air contaminants. The focus is on imputation methods to deal with missing data and verify if the use of synthetic data can improve the ML predictions. The interpretability assessment of the ML model, by use of Shapley Additive Explanation (SHAP), provided insights into the influence of experimental conditions on the TiO_2_ photocatalytic performance. The SHAP analyses aids in understanding the connections between different factors, thereby could facilitate informed and efficient decision-making in the design of air contaminants removal.

## Methodology and technical background

**Figure 1:**
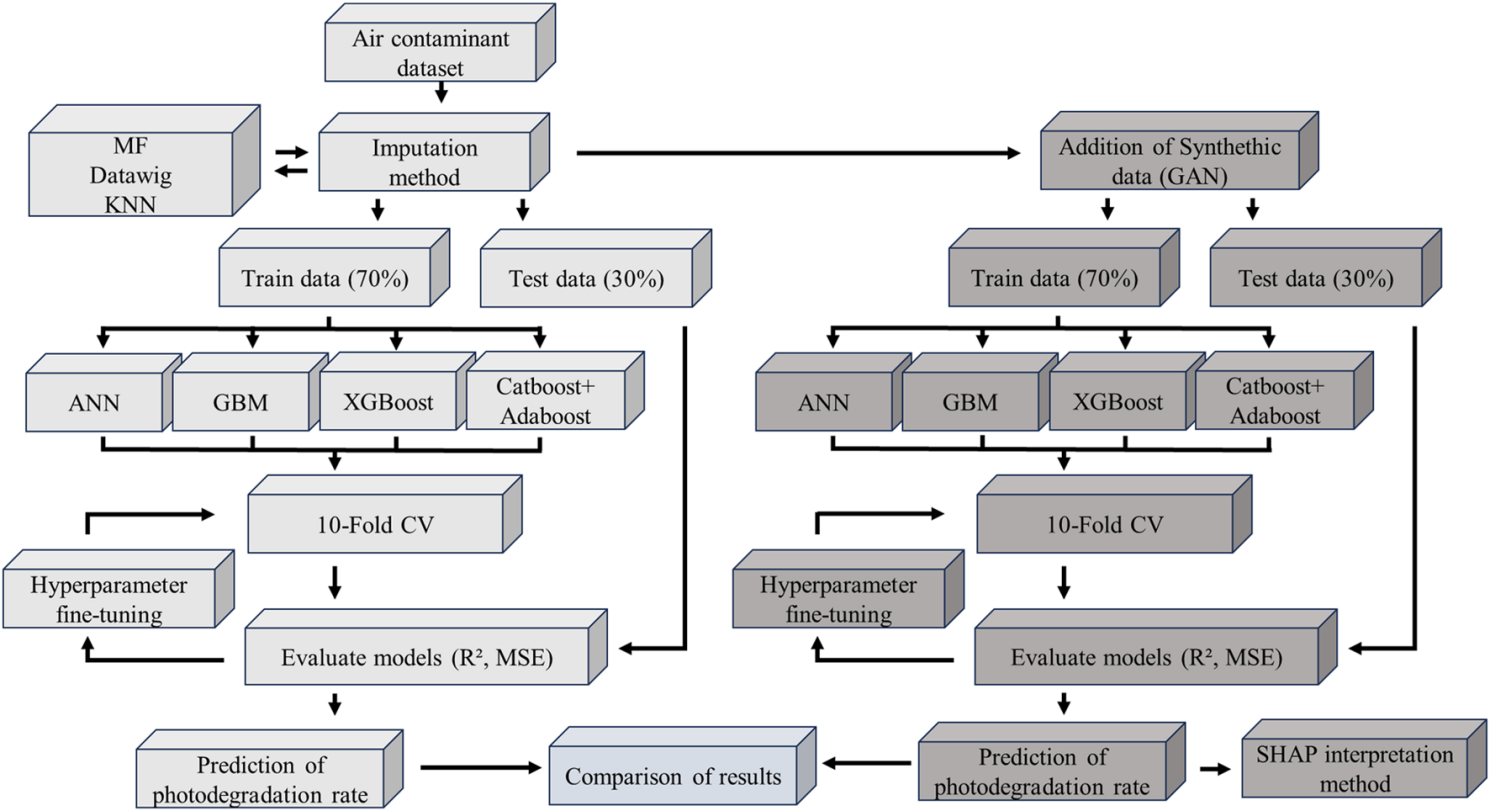
Schematic of the analysis’s procedures used in this study.

The schematic of investigation procedures applied in this research is shown in Fig. 1. Four different types of ML models are developed, including an artificial neural network (ML) and gradient booster ML model, both optimized with Bayesian optimization, XGB model with hyperparameters optimization using HYPEROPT and Catboost model combined with Adaboost. An experimental dataset was collected from the published literature and used in the analyses to predict the photocatalytic-degradation performance of TiO_2_ in removing air-born contaminants. Synthetic data is also used for the analysis. The performance of different models is compared. The influence of factors on photocatalytic performance is evaluated quantitatively using the SHAP method.

### Data collection, processing and augmentation

The database used for ML model development consists of 200 sets of experimental data which are obtained from published papers^27–52^. The comprehensive dataset is shown in Table S1 in the supplementary information. This dataset has eight design features, i.e., the type of air-born organic contaminant (OC), ultraviolet light intensity (I, mW/cm^2^), wavelength (W, nm), dosage (D, mg/cm^2^), humidity (H, %), experimental temperature (T, °C), reactor volume (R, L), initial concentration of air contaminant (InitalC, ppmv), which essentially covers most important settings of experimental conditions. The photo-degradation rate (k, min^−1^/cm^2^) is set as the response variable for the ML model output. Since the absolute value of reaction rate is typically a small positive number, it is transformed into base 10 logarithm − log(k) to amplify small values.

All the variables, except for the air contaminant type, along with the output response, are numerical data and can be immediately comprehended by computational linguistics. Molecular fingerprinting was implemented to transform the air contaminant type into a readable binary vector. Molecular fingerprints present a way to record each molecule in a mathematical form, enabling machine learning and computational programming to be performed on chemical data. The creation of molecular fingerprints aims to identify substructures and measure molecular resemblance in extensive databases. Recent studies have utilized this technique to describe tiny molecule structural variables in machine learning^53–55^. When a molecule is fingerprinted, it is transformed into a binary vector that can be learned by the machine learning model. The vector provides information about each atom's molecular properties, such as its atomic number, charge, mass, attached hydrogen, and any bonding neighbors it has that are not hydrogen^56^.

The air pollutants are initially modified and converted into two-dimensional SMILES (simplified molecular input line entry system) strings. SMILES is a shorthand notation system that uses simple ASCII characters to represent organic molecules and their structures, enabling connections between chemical knowledge and computer programming.

Molecular fingerprints (MF) have been found to be effective in quantitative structure–activity relationship (QSAR) research and in developing predictive machine-learning models^57–60^. The molecule's variables could be easily read, and the model demonstrated a reasonable level of prediction accuracy. The "AllChem.GetMorganFingerprintAsBitVect()" function is used for the models. After transformation, a binary digit vector, such as (1 0 1…. 0), was used to describe the water contaminants. The length of the resulting binary fingerprint vector depends on the choice of nBits. Increasing the value of nBits results in longer fingerprints that may contain more information but also leads to higher computational complexity. In the study, a value of 1024 for nBits was used, based on experimentation and optimization, to strike a balance between computational efficiency and predictive performance. For the radius, a value of 3 was selected, as higher radius encodes larger fragments.

In Table 1, the statistical features for the dataset, comprising both input and output variables used for ML models, are presented. Additionally, the table indicates the extent of missing values for certain experiments.

**Table 1:** Summary of the characteristics of dataset on TiO_2_ photocatalytic degradation of air-born organic contaminants.

Variables	Min	Max	Mean	SD	Missing value (%)
Inputs					
Organic contaminant (smile)	Organic molecules encoded with SMILES				
Intensity (mW/cm^2^)	0.36	80.00	10.00	14.89	17.50
Wavelength (nm)	0.00	565.00	358.00	68.34	11.50
Dosage (mg/cm^2^)	0.01	96.00	0.40	19.48	32.00
Humidity (%)	0.00	1600.00	40.00	147.42	13.50
Temperature (°C)	22.00	350.00	45.00	42.82	27.50
Reactor (L)	0.04	216.00	0.89	39.47	4.50
Initial Concentration (ppmv)	0.00	5944.00	20.00	1384.94	9.50
Output					
Photo-degradation rate (min^−1^/cm^2^)	0.000	0.037	0.001	0.005	13.00

#### Missing data imputation

Some features are missing in specific experimental samples. From our observations, elements with multiple missing data can significantly influence the quality of ML predictions. Therefore, imputations are used to fill the missing values as a powerful alternative to removing the dataset, which is crucial given the relatively small size of the dataset commonly encountered in engineering research. In this study, three different imputation methods were used: Imputation using MissForest, Datawig, and K-Nearest Neighbors (KNN), as demonstrated in Fig. 1. The values obtained were compared, and the best imputation method was determined. The principles of these imputation methods are explained below.

##### Imputation using MissForest (MF)

Breiman's random forest serves as the foundation for MissForest, an iterative imputation technique^61^. Random forest is a machine learning technique that involves averaging multiple classification or regression trees without pruning. This approach effectively creates a system for multiple imputation, with several desirable properties, including its ability to handle complex interactions and nonlinearity as a non-parametric method. Additionally, it can deal with different types of missing data and adjust easily to high-dimensional data. Even without prior knowledge of the original data, random forest produces excellent imputation results.

##### Imputation using Datawig

DataWig’s SimpleImputer analyzes data and fills missing values autonomously using deep learning. The model functions by establishing an output feature and input features consisting of data that can aid in computing the determined output element. This method is capable of imputing values for numeric, non-numeric, and heterogeneous data types^62^. Datawig is implemented as a Python library for replacing missing features in a dataset. An open-source package containing the code for this approach is available at https://github.com/awslabs/datawig.

##### Imputation using K-Nearest Neighbors (KNN)

The K-Nearest Neighbors (KNN) imputation method^63^ is a technique that involves calculating the distance between data points to establish k neighbors, whose arithmetic mean is then used to estimate the missing values. This imputation method can be applied to continuous, discrete, ordinal, and categorical data, making it a suitable solution for all types of missing data^64^. To implement KNN imputation, the KNNImputer class algorithm supported by Scikit-Learn is used, which inserts missing values by using the mean estimates of the KNN.

#### Synthetic Tabular data

Generative Adversarial Networks (GANs) were initially established by Goodfellow^65^ and can be considered a Neural Network algorithm for generative modeling. These models are based on a two-player game theoretical scenario, aiming to produce fresh data with characteristics similar to the initial training set while explaining patterns and distributions in the training data^66^.

In this study, a type of GAN called "vanilla GAN"^62^ was utilized. A vanilla GAN consists of a generator (G) and a discriminator (D). The generator takes input from a multivariate Gaussian distribution and produces new data, while the discriminator's job is to determine whether the created data is similar to the actual training samples. The mathematical definition is derived from the cross-entropy between real and created distributions. This process involves two deep learning architectures set up in a minimax configuration, as shown in Eq. (1)

1 $$\underset{G}{\text{min}}\underset{D}{\text{max}}V(D, G)={E}_{x\sim {p}_{data}(x)}[\text{log}D(x)]+{E}_{z\sim {p}_{z}(z)}[\text{log}(1-D\left(G\left(z\right))\right)]$$

In Eq. (1), the discriminator D(x) seeks to maximize the quantity $V(G,D)$ for any provided generator $G\left(z\right)$, where $G\left(z\right)$ represents the generator’s output when given z. ${E}_{x\sim {p}_{data}(x)}$ and ${E}_{z\sim {p}_{z}(z)}$ represent the expected values over all real data occasions and over all generated fake occasions, respectively. When ${p}_{data}={p}_{g}$, the task reaches the global optimum, analogous to the global minimum of the training criterion. The discriminator D and generator G must be jointly optimized to lower the possibility of overfitting when training finite datasets. At the start of the learning process, the discriminator D may reject the high-confidence samples developed by the generator G due to differences compared with samples from the training data. For this reason, instead of decreasing $\text{log}(1-D\left(G\left(z\right)\right)$ for the generator G, it can be trained to increase $\text{log}(D\left(G\left(z\right)\right)$. When the generator and discriminator are perfect, they reach a Nash equilibrium where the generator is creating an identical data distribution, and the discriminator is unable to differentiate between the two distributions.

### Machine learning (ML) models

The overview of the employed ML models in this study and respective optimization methods can be seem in the supplementary section.

#### ML interpretability: SHapley additive explanations (SHAP)

The Shapley value method, rooted in the principles of cooperative game theory, offers a versatile approach for assigning importance rankings to individual attributes within classification examples, providing valuable insights for decision-making. This concept finds practical application in machine learning, particularly in the evaluation of model predictions. Lundberg and Lee^67^ have highlighted the potential of SHAP, wherein input variables are analogized as 'players' contributing to a 'payout' in a game-like scenario. SHAP allows us to quantitatively assess the unique contributions of each 'player' to the overall prediction, helping to unveil the inner workings of machine learning models both at a global and local scale^68^.

SHAP serves as a valuable tool for interpreting machine learning model outputs, ensuring consistency in its feature attributions. It calculates Shapley values for each feature, indicating their impact on the model's predictions when included, whether positively or negatively^69^. Furthermore, SHAP is accessible through a Python toolkit designed for transparent machine learning, making it a practical choice for understanding complex models and datasets^70^.

#### Parameters for ML model performance assessment

To determine the effectiveness of the created models, two parameters were used to evaluate their performance in solving regression problems. The first parameter, referred to as the correlation coefficient (R^2^), measures the closeness of the data to the fitted regression line or the percentage of the response variable change described by a linear model. R^2^ normalizes the squared residual error with the database's variance and gives a score ranging from 0 to 1. The second parameter, mean squared error (MSE), measures the degree of error in statistical models by computing the average squared difference between actual and predicted values. Equations (2) and (3) show these two parameters.

2 $${R}^{2}=\frac{{[{\sum }_{i=1}^{n}({y}_{i}-\underline{y})({{y}{\prime}}_{i}-\underset{\_}{y{\prime}})]}^{2}}{{{\sum }_{i=1}^{n}{({y}_{i}-\underline{y})}^{2}\cdot {\sum }_{i=1}^{n}({{y}{\prime}}_{i}-\underset{\_}{y{\prime}})}^{2}}$$

3 $$MSE= \frac{{\sum ({y}_{i}-\widehat{{y}_{i}})}^{2}}{n}$$

In these equations, the symbols ${y}_{i}$ and ${y{\prime}}_{i}$ represent a real value and its corresponding predicted value, respectively. The symbols $\underline{y}$ and $\underset{\_}{y{\prime}}$ denote the averages of the actual and predicted values, respectively. These are used to quantify the discrepancy between the actual and predicted values.

## Results and discussions

In this section, the performance of imputation techniques was assessed by analyzing the R^2^ and MSE values computed from various ML models. The imputation approaches utilized in this investigation are MF, Datawig, and KNN. Additionally, the dataset with additional synthetically generated data is also evaluated in a similar way. Finally, the SHAP analysis is conducted to obtain the feature importance of the inputs.

### Performance of the models with different data imputation methods for missing data

With data imputation methods used to estimate missing data, the comparison of the performance of various machine learning techniques used in this study to estimate the rate of photo-degradation of air contaminants is presented in Table 2. The models were evaluated using both MSE and R^2^ values for both the training and test sets. For the initial split of the data for all models, 70% of the data is used for training and developing the machine learning model, while the remaining 30% is reserved for the test set. The training and testing data are randomly selected. The training set evaluation measures the model's ability to interpolate established data, while the test set evaluation assesses the accuracy of the model's predictions on independent data. Across all the models used, the R^2^ values were close to 1, while the MSE values showed slight variations. To obtain accurate and reliable predictions, the choice of model should be based on a combination of high R^2^ and low MSE results^71^. Additionally, a model with extremely high values for the training set and lower values for the test set may be overfitted, while a good combination of results for both sets indicates a stronger model. Therefore, models should aim to balance complexity and interpretability to achieve a high degree of accuracy.

**Table 2:** Performance comparison for prediction of photo-degradation rate of air contaminants from various ML Techniques.

Model	HYPEROPT method for XGB	R^2^		MSE		Imputation method
		Train	Test	Train	Test	
ANN with BO		0.976	0.577	0.024	0.318	MF
Catboost + adaboost		0.996	0.903	0.004	0.066	
GBM with BO		0.997	0.835	0.003	0.124	
XGB with HYPEROPT	Random search	0.992	0.823	0.008	0.133	
	Annealing	0.983	0.805	0.017	0.146	
	TPE	0.993	0.831	0.007	0.127	
ANN with BO		0.964	0.087	0.034	0.602	Datawig
Catboost + adaboost		0.996	0.736	0.004	0.174	
GBM with BO		0.995	0.362	0.004	0.420	
XGB with HYPEROPT	Random search	0.996	0.482	0.004	0.342	
	Annealing	0.976	0.498	0.023	0.331	
	TPE	0.996	0.482	0.004	0.342	
ANN with BO		0.932	0.438	0.079	0.559	KNN
Catboost + adaboost		0.971	0.922	0.034	0.064	
GBM with BO		0.971	0.882	0.034	0.117	
XGB with HYPEROPT	Random search	0.970	0.910	0.035	0.089	
	Annealing	0.963	0.915	0.043	0.085	
	TPE	0.971	0.927	0.033	0.073	

Based on the results presented in Table 2, KNN imputation is considered the best option for all models except ANN when considering the balance between train and test set metrics. This is demonstrated, for example, when considering the XGB model with the random search algorithm, which achieved an R^2^ value of 0.970 for training data and 0.910 for testing data using KNN imputation, with acceptable balance between train and test sets. However, this doesn’t happen when compared with the same model using MF and Datawig imputation methods. The train set R^2^ metrics for these models are 0.992 and 0.996, respectively, while the test set R^2^ metrics are 0.823 and 0.482. This difference between train and test sets indicates overfitting of the models, suggesting that they learn the original data very well but struggle with new data.

Following this pattern, the combination of XGB with HYPEROPT using the TPE algorithm and KNN imputation method produced the best results when comparing the performance of all types of machine learning models. The R^2^ value for the training set was 0.971, while the R^2^ for the testing set was 0.927, and the MSE value for the training dataset was 0.033, and for the testing dataset, it was 0.073. These results indicate that the model was able to make very good predictions for the photo-degradation rate of air contaminants.

**Figure 2:**
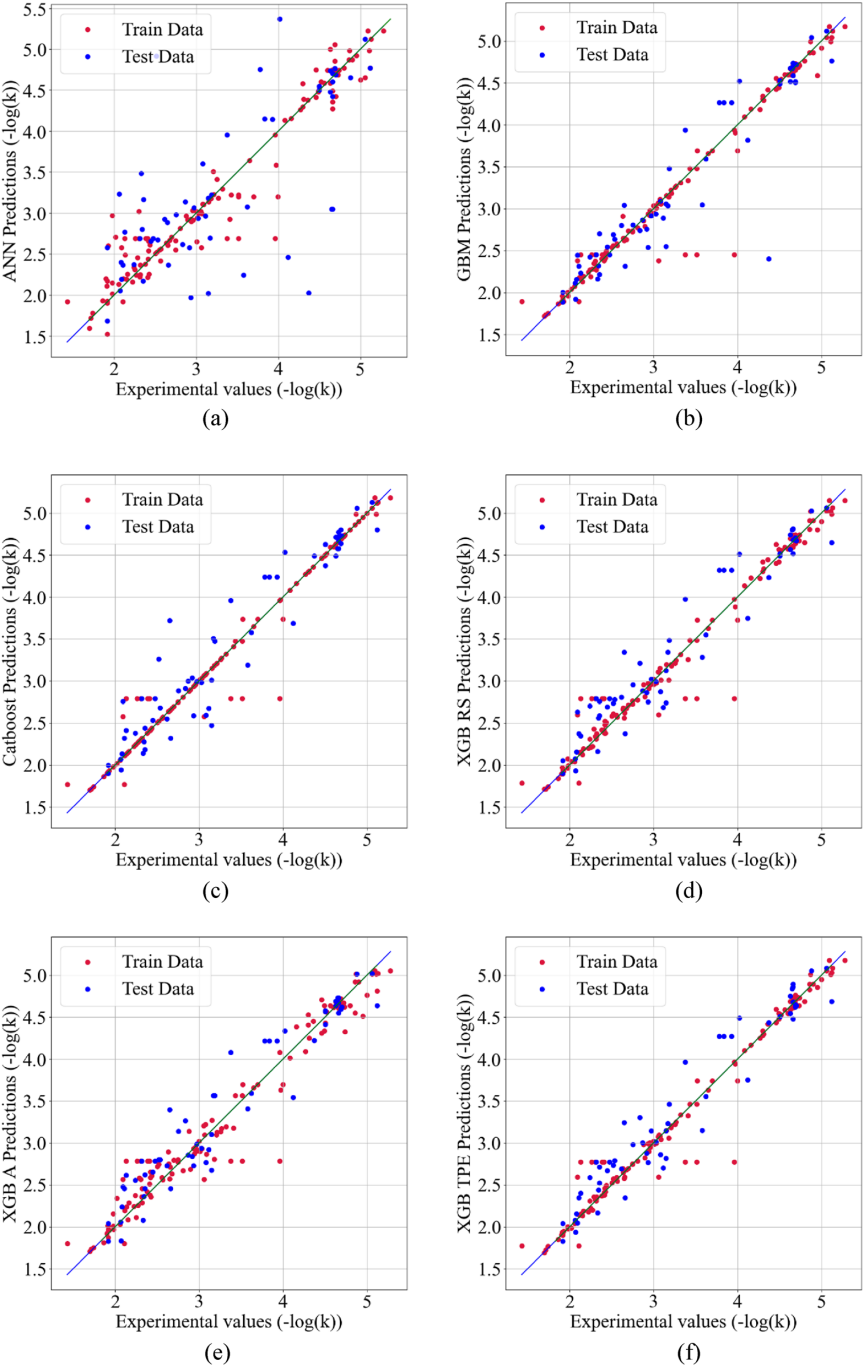
The scatter plots of the predicted vs. experimental photo-degradation rate constants –log(k) using KNN imputation for (**a**) ANN, (**b**) Catboost + adaboost, (**c**) GBM, (**d**) XGB Random search, (**e**) XGB Annealing, and (**f**) XGB TPE.

Figure 2a–f show scatter plots of the predicted vs. actual photo-degradation rate constants (− log(k) values) for the training and testing sets using KNN imputation for ANN, Catboost, GBM, XGB with RS, XGB with Annealing, and XGB with TPE, respectively. The strong correlation between the measured and predicted photodegradation rate for all the models except ANN serves as evidence of the models' good predictive capabilities and indicates their good performance. In the supplementary section, Figs. S2 and S3 display scatter plots of the predicted vs. actual photo-degradation rate constants (− log(k) values) for all models using MF and Datawig imputation, respectively.

The catboost + adaboost model achieved similar results to the best model, with an R^2^ value of 0.971 for the training dataset and an R^2^ value of 0.922 for the testing dataset. However, the training and testing datasets had different MSE values, with the training dataset having an MSE of 0.034 and the testing dataset having an MSE of 0.064. The other models that used KNN imputation also performed exceptionally well, except for the ANN model.

As discussed, the KNN, XGB with HYPEROPT using TPE algorithm, and catboost + adaboost models demonstrated very similar results. When using the MF and Datawig imputation methods, catboost + adaboost achieved better results than ANN, XGB, and GBM. These findings suggest that using an ensemble method and combining models can be an effective approach for achieving superior results. However, the GBM and ANN models showed indications of overfitting based on the differences between their train and test results for R^2^ and MSE. While the results were acceptable for models using MF imputation, there were still some signs of overfitting, particularly for the ANN and GBM models. In contrast, the results for models using the Datawig imputation method exhibited significant overfitting, which could be attributed to the limited number of samples in the dataset and the high-variance nature of Datawig. To address this issue, the next section of the study will investigate whether synthetically generated data can reduce overfitting and improve the results.

### Performance of the models with synthetic data generated

#### Data synthetically generated

**Figure 3:**
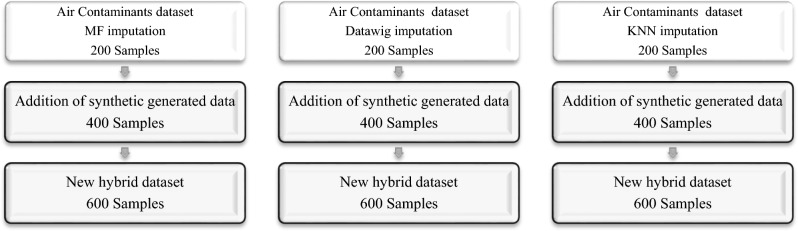
Procedure for synthetic data.

**Figure 4:**
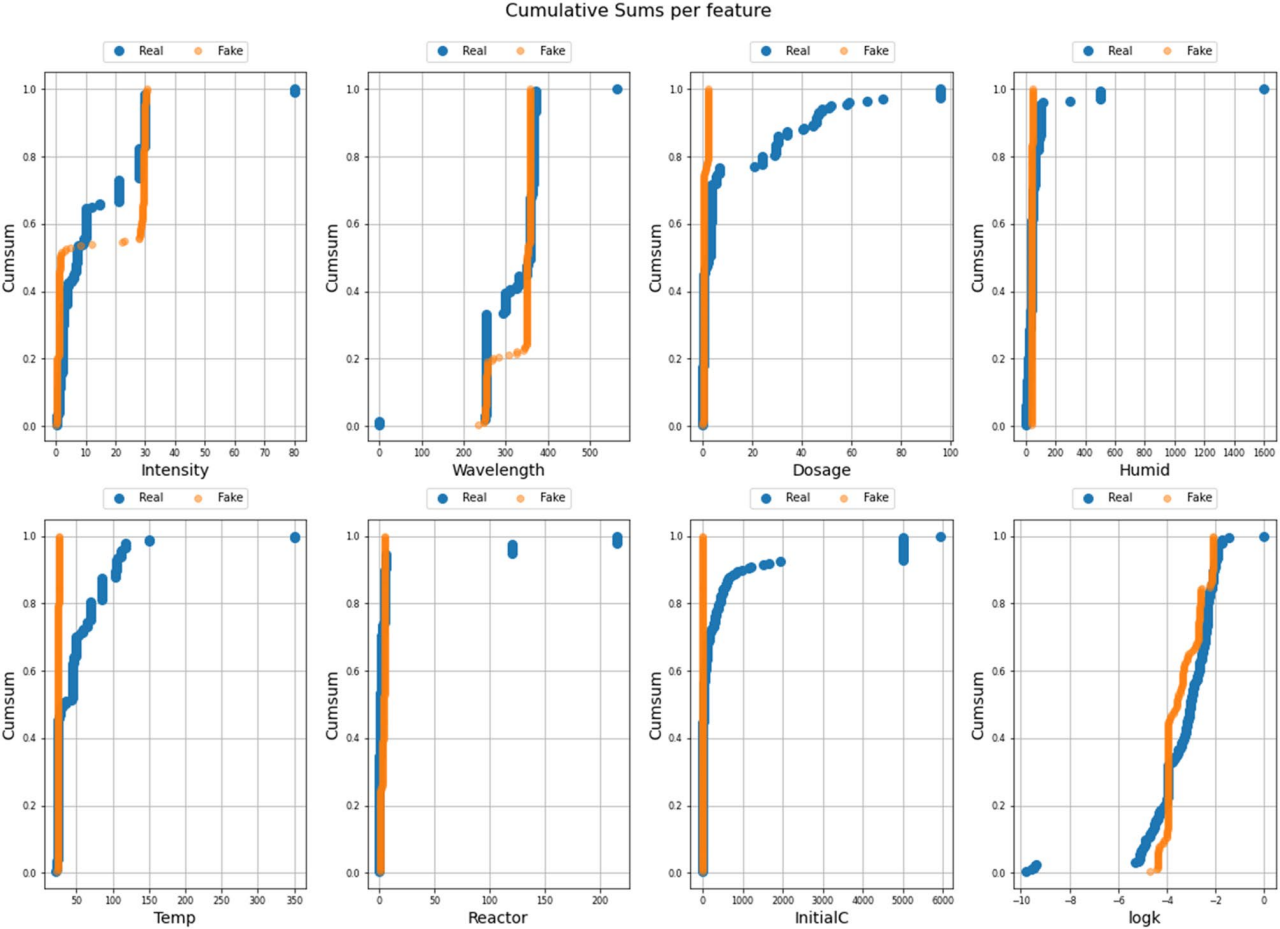
Comparison between real and generated fake samples.

The synthetically generated samples used in this research were generated with Vanilla GAN. For the implementation of the Vanilla GAN, a Python package called ydata-synthetic^72^ was utilized. The addition of synthetic data followed the procedure demonstrated in Fig. 3. The original dataset contained 200 samples with imputed data using the MF, Datawig, and KNN methods, and 400 additional samples were generated and added to each imputation model. This resulted in a total of 600 samples imputed with each method. Table 3 displays the generator and discriminator parameters and layers used for MF imputation, including the batch size and dimensions of each layer. For example, an output layer of (32, 128) represents 32 for the batch size and 128 for the dimension. Other parameters include log step = 100, epochs = 10,001, and a learning rate of 5e-4. The best architecture was selected after a search with several trials to obtain the architecture that would yield samples with behavior very similar to the real ones. After the adoption of the best architecture, Fig. 4 depicts a comparison of real and synthetic images, with a cumulative sum per feature plot aiding in understanding how each feature contributes to the overall cumulative sum and how similar the original and synthetic data are.

**Table 3:** Generator and discriminator parameters of the GAN.

Generator			Discriminator		
Layer type	Output shape	Parameters	Layer type	Output shape	Parameters
Input layer 1	(32, 32)	0	Input layer 2	(32, 15)	0
Dense layer 1	(32, 128)	4224	Dense layer 5	(32, 512)	8192
Dense layer 2	(32, 256)	33024	Dropout layer 1	(32, 512)	0
Dense layer 3	(32, 512)	131584	Dense layer 6	(32, 256)	131,328
Dense layer 4	(32, 15)	7695	Dropout layer 2	(32, 256)	0
–	–	–	Dense layer 7	(32, 128)	32,896
–	–	–	Dense layer 8	(32, 1)	129

#### Performance of ML model trained with augmented synthetic data

**Figure 5:**
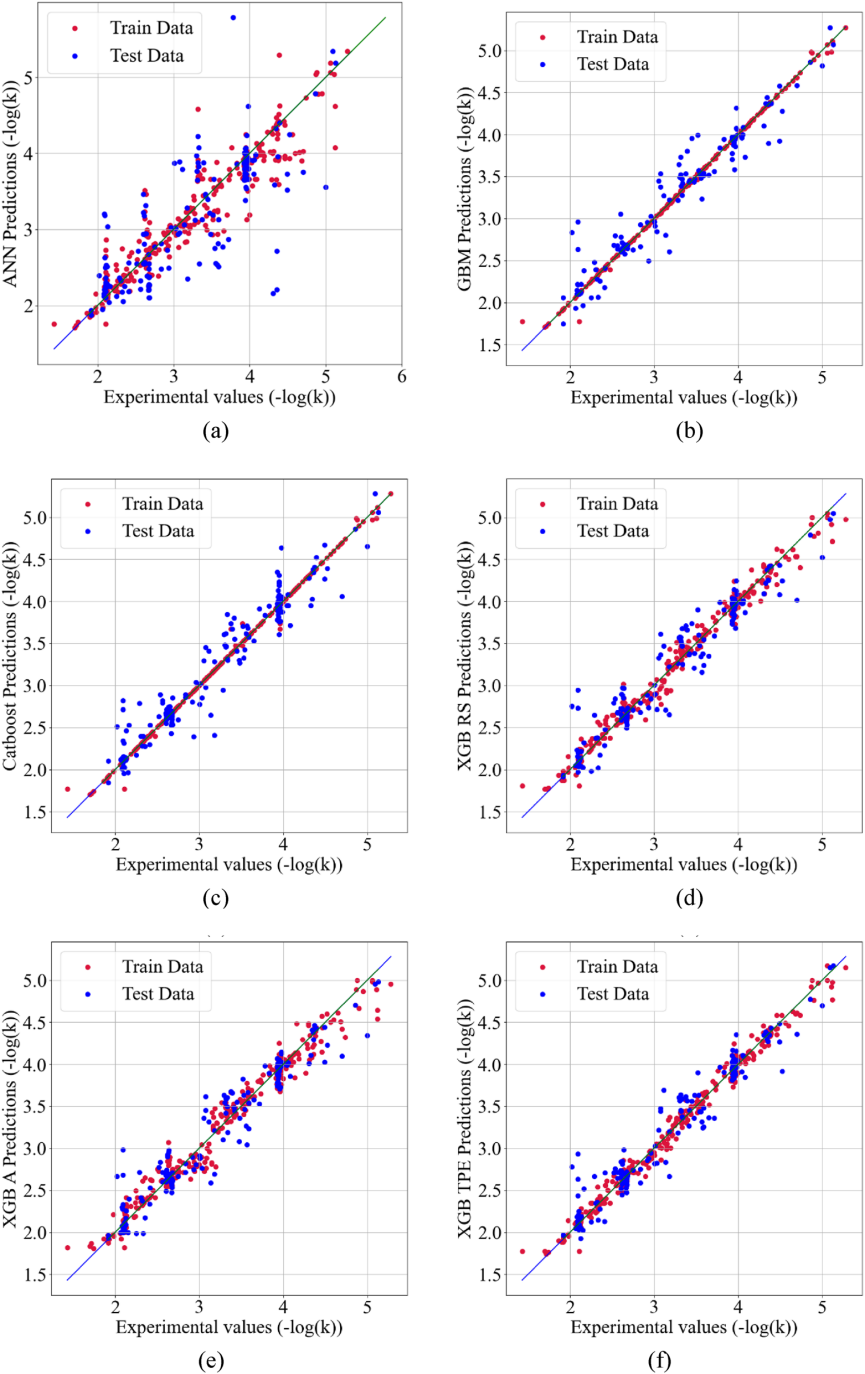
The scatter plots of the predicted vs. experimental photo-degradation rate constants –log(k) using MF imputation AND synthetic data for (**a**) ANN, (**b**) Catboost + adaboost, (**c**) GBM, (**d**) XGB Random search, (**e**) XGB Annealing, and (**f**) XGB TPE.

The number of samples in the dataset was increased three-fold by generating synthetic data using Vanilla GAN, resulting in a total of 600 samples. The four ML models were then trained using the synthetically generated data for predicting the photo-degradation rate of air contaminants. The performance of each ML model is presented in Table 4, where it is shown that, except for the ANN model, all ML models improved their performance with increased R^2^ values and decreased MSE errors. The models with MF imputation performed the best overall, followed by KNN and Datawig imputation models. The addition of synthetic data significantly reduced overfitting for all models. The scatter plots of the predicted vs. experimental photo-degradation rate constants, –log(k), for each model are presented in Fig. 5a–f, showing scatter plots of the predicted vs. actual photo-degradation rate constants (− log(k) values) for the training and testing sets using MF imputation for ANN, Catboost, GBM, XGB with RS, XGB with Annealing, and XGB with TPE, respectively. The strong correlation between the measured and predicted photodegradation rate for all the models except ANN serves as evidence of the models' good predictive capabilities and indicates their strong performance.

**Table 4:** Performance comparison for prediction of photo-degradation rate of air contaminants from various ML Techniques with synthetic data.

Model		R^2^		MSE		Imputation method
		Train	Test	Train	Test	
ANN with BO		0.900	0.573	0.069	0.265	MF
Catboost + adaboost		0.999	0.961	0.001	0.003	
GBM with BO		0.992	0.935	0.005	0.040	
XGB with HYPEROPT	Random search	0.982	0.944	0.012	0.035	
	Annealing	0.995	0.944	0.003	0.035	
	TPE	0.996	0.940	0.003	0.037	
ANN with BO		0.736	0.392	0.342	0.156	Datawig
Catboost + adaboost		0.997	0.899	0.002	0.059	
GBM with BO		0.997	0.900	0.002	0.056	
XGB with HYPEROPT	Random search	0.996	0.924	0.002	0.043	
	Annealing	0.994	0.928	0.004	0.041	
	TPE	0.996	0.927	0.002	0.041	
ANN with BO		0.932	0.708	0.052	0.214	KNN
Catboost + adaboost		0.983	0.927	0.013	0.056	
GBM with BO		0.985	0.900	0.011	0.073	
XGB with HYPEROPT	Random search	0.978	0.928	0.017	0.053	
	Annealing	0.986	0.935	0.011	0.048	
	TPE	0.978	0.928	0.017	0.053

In the supplementary section, Figs. S4 and S5 display scatter plots of the predicted vs. actual photo-degradation rate constants (− log(k) values) for all models with synthetic data using KNN and Datawig imputation, respectively. The Catboost + adaboost model with MF imputation performed the best, with a train R^2^ value of 0.999 and a test R^2^ value of 0.961, while the train MSE value was 0.001 and the test MSE value was 0.003. Two other models, XGB with HYPEROPT using MF imputation and the annealing algorithm, and XGB with HYPEROPT using MF imputation and the TPE algorithm, also performed well. For models with Datawig and KNN imputation, XGB with HYPEROPT showed the best metrics. Overall, all models showed improved R^2^ and MSE values and reduced overfitting with the addition of synthetic data, except for the ANN model, which didn’t improve the metrics as much as the other models. Further explorations are needed to determine the reason for the lack of improvement in the performance of the ANN model trained with synthetic data compared to other models.

#### Analysis and comparison of models with regular and synthetic data

When comparing the behavior of regular data and synthetic data, based on the results established before by Tables 2 and 4 where the KNN imputation method performed best for regular data, while the MF imputation method performed best for synthetic data, it is possible to note that both methods delivered excellent results for most models. When comparing the metrics for R^2^ (train and test) and MSE (train and test) for all models using MF imputation and KNN imputation with the models using synthetic generated data, it is possible to see that R^2^ values increased for both train and test datasets for almost all models, except for the ANN model. The Catboost + adaboost model showed the best improvement, with the test set increasing from 0.903 to 0.961, while the train set had a small increase going from 0.996 to 0.999. The addition of synthetic data significantly reduced the MSE error for all models except the ANN model, particularly for the test set. Overfitting was also significantly reduced for most models, except for the ANN model. For the best model, Catboost + adaboost, the MSE for the train set decreased from 0.004 to 0.001, while the test set had a decrease from 0.066 to 0.003. Especially in the test set, the error decreased by more than 50% in most cases. Furthermore, adding the synthetic data significantly reduced the overfitting in these ML models, except for the ANN model.

For missing data estimation using KNN imputation, when comparing metrics, a lower increase in the R^2^ for all the models except ANN, compared to the MF imputation method, can be seen. The MSE error was reduced for all the models, except ANN. This again shows that adding synthetic data improved at least a little for these ML model performances and reduced overfitting.

Overall, the observations show that the accuracy of ML models improved significantly with the use of the synthetic data, which is twice the size of the original dataset in this case. This provides a promising and effective way to improve ML model performance with a limited amount of dataset. However, the ANN model improved to a lesser extent than the other models.

### SHAP Interpretability of the trained ML models

The previous analyses show that the CatBoost + AdaBoost model with MF imputation yielded the best results in predicting the photo-degradation rate of air contaminants using original data augmented with a synthetic dataset. To shed light on the interpretability of this optimal ML model, the importance factors of each input variable are analyzed. The importance factor indicates how much an input variable affects the accuracy of ML model prediction. The larger the importance factor of an input variable, the more important its effect on ML model prediction.

Here, the SHAP (SHapley Additive exPlanations) framework^73^ is used to quantify the importance factors of the experimental variables studied^59^. Basically, SHAP values are calculated by comparing two models with and without the inputs. The predictions from the two models are compared, and the effect of the input on the prediction is determined. A higher average SHAP value indicates a higher magnitude (positive or negative) and is more influential. These analyses help understand the effects of the experimental variables on the performance of the photocatalytic degradation process, thus offering guidance for practical design in real applications.

**Figure 6:**
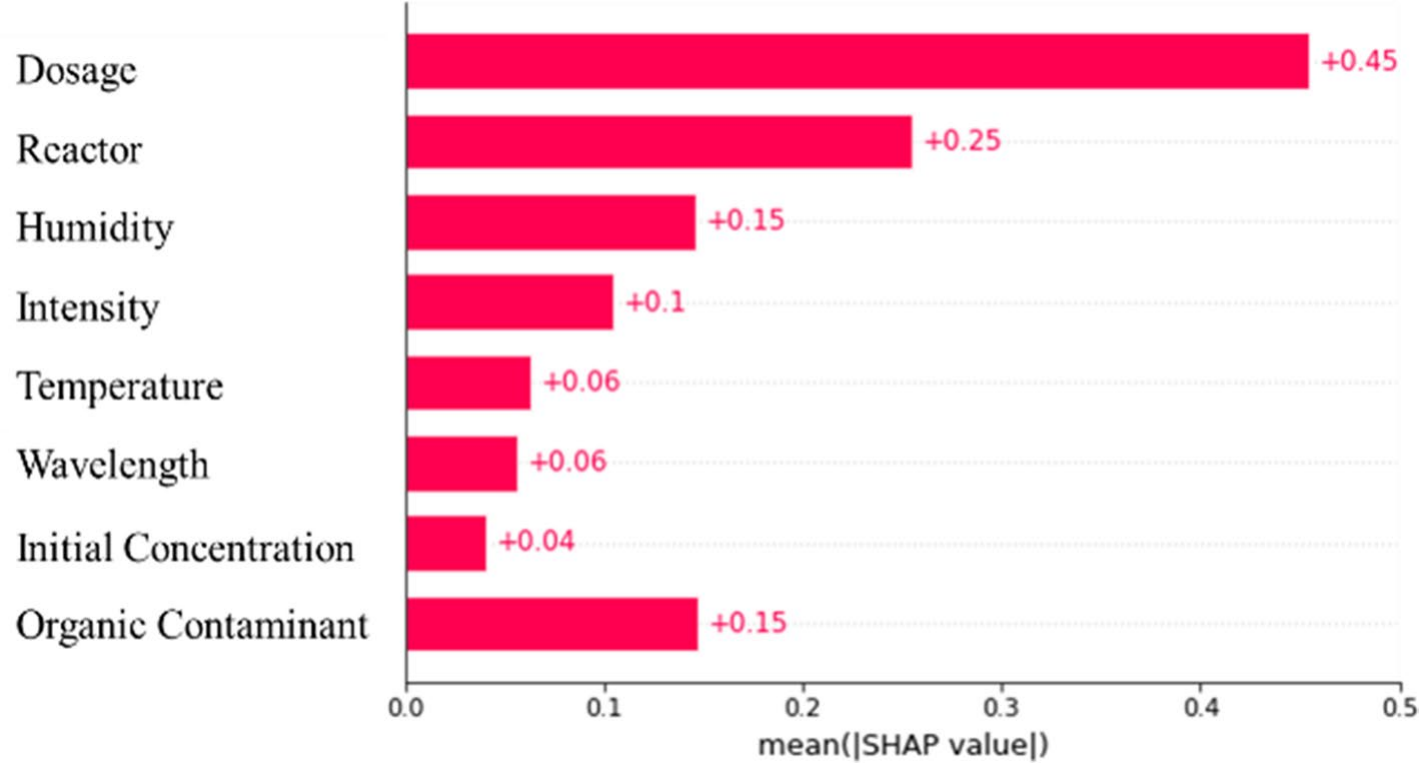
The bar plot of the importance factors (mean SHAP values) of each variable on TiO_2_ photo-degradation rate of air contaminants.

For an initial global analysis, Fig. 6 shows the average of the mean SHAP value of different input variables on the photo-degradation rate predictions. The figure shows that the dosage of photocatalyst has the largest SHAP value of 0.45, which means it has the greatest influence on the photo-degradation rate of air contaminants. Reactor size followed with a mean SHAP value of 0.25. Humidity and the type of organic contaminant both have a value of 0.15. Light intensity had a mean SHAP value of 0.1, while temperature and wavelength have SHAP values of 0.06. The initial concentration of contaminant has an SHAP value of 0.04.

The relatively larger SHAP value for reactor size implies that the photocatalytic reaction by TiO_2_ in the decomposition of airborne contaminants is diffusion-controlled. This is consistent with the results of experimental observations^69^. The small SHAP value for the initial concentration of contaminants implies that the reaction is likely to be a first-order reaction. Relative humidity plays an appreciable role in the photocatalytic reaction rate, possibly due to its ability to form dissolved oxygen species that enhance photocatalytic reactions.

**Figure 7:**
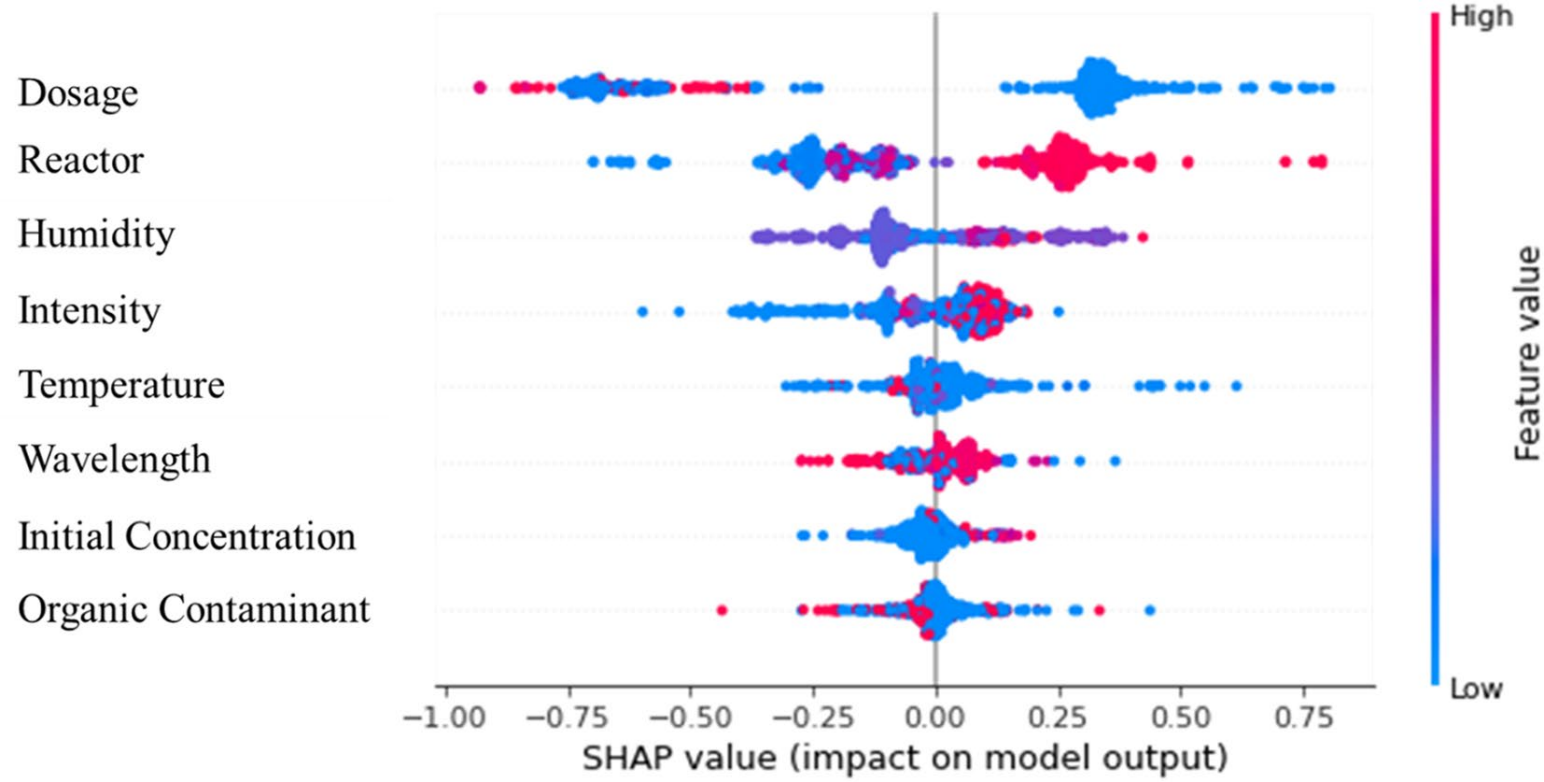
Beeswarm density scatter plot of SHAP values of features affecting photocatalytic reaction rate of TiO_2_ in air contaminants degradation.

The Beeswarm plot is used to further indicate how the change of input variables affects the model predictions. Figure 7 generates this plot that merges the importance of the variable with variable effects for the CatBoost + AdaBoost model. Every individual point in the plot represents a SHAP value for a variable and an occurrence. Figure 7 shows the SHAP values of all test datasets for predicting the photocatalytic degradation rate, where features listed on the left are sorted by the average SHAP value for all test samples with the average magnitude listed in Fig. 6. In the row of each feature, the points indicate the corresponding SHAP values with the magnitude measured by the horizontal ordinate and the color representing the input value of that individual (red for high, blue for low). For example, Dosage is a continuous feature with magnitudes ranging from 0.01 to 96.00 mg/cm^2^ and is coded by color. Experimental data with larger dosages of photocatalyst are indicated in red, while those with smaller dosages are indicated in blue. Meanwhile, the points on the left side of the midline make the ML model predicted reaction rate lower, while on the right side they make the predicted reaction rate higher. The farther away from the midline, the greater the impact on the predictions.

From Fig. 7, it can be generally observed that lower values of dosage have positive SHAP values, considering that the points extending towards the right are increasingly blue, while some high values of dosage have negative SHAP values moving towards the left. This indicates that air contaminants with lower dosages have higher predicted photo-degradation rates, while higher dosages have lower predicted photo-degradation rates. This might indicate the possible effects on the clustering of photocatalysts. Higher dosage makes it more likely to form clusters, which compromises the photocatalytic reaction rate per unit dosage, and vice versa.

For the reactor size, which ranges from 0.04 to 216.00 L in the dataset assembled in this study, larger reactor sizes have positive SHAP values as large as 1, while small reactor sizes have negative SHAP values as low as around − 0.75. This indicates that a larger reactor size contributes to the increase of the photocatalytic reaction rate. Similarly, for humidity, Fig. 7 shows that larger humidities generally correspond to positive SHAP values (or larger predicted photocatalytic reaction constants), while lower humidities correspond to negative SHAP values. This indicates that humidity positively affects the photocatalytic reaction rate. For light intensity, Fig. 7 shows a dense cluster of high and low instances with small but positive SHAP values from 0 to around 0.25. Instances of low-intensity values extend further towards the left, reaching SHAP values of − 0.75. This suggests that lower light intensity has a stronger negative impact on the photo-degradation rate.

Following a similar interpretation, for the remaining features, which have a less significant impact on the predicted reaction rates, general trends of their influence can be obtained from Fig. 7. In summary, increasing light wavelength negatively affects the reaction rate, which is due to lower energy with larger light wavelengths; increasing temperature negatively affects the photocatalytic reaction rate, possibly due to high temperatures reducing water vapor absorption by photocatalysts to form dissolved reactive oxygen species. A higher initial concentration of the contaminants corresponds to higher reaction rates. These are consistent with what's expected from physical observations. Therefore, the SHAP analyses, including the Beeswarm plot, provide interpretability of the high-performing ML model (CatBoost + AdaBoost model with MF imputation) on the datasets.

**Figure 8:**
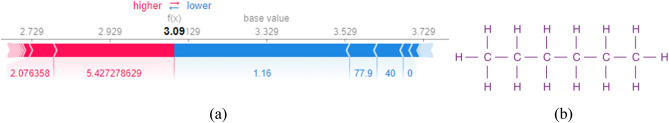
Hexane (**a**) SHAP force plot and (**b**) structure.

The organic contaminant feature in Fig. 7 combines all contaminants and produces both positive and negative effects. This effect can't be understood without knowing the composition of each element. The SHAP method considers how each component affects the prediction based on the chemical structure of each element. When converted using SMILES, they are divided into features. For example, in Fig. 8a, the SHAP force plot shows the effect of hexane based on its structure shown in Fig. 8b. The binary features, 0 and 1, each have an impact calculated by the SHAP method to influence the predictions.

## Conclusions

This study investigated the prediction of the photo-degradation rate constant of TiO_2_ for air contaminants using four different types of ML models: ANN, GBR, XGB, and Catboost + Adaboost. A data collection protocol was implemented where an experimental dataset was assembled from published literature. Data quality assessment indicates that a large portion of published data lacked certain factors affecting the photocatalytic reaction rates. In light of this, imputation methods were applied to estimate the missing values to evaluate the performance of all ML models.

All trained ML models were found to achieve good performance in predicting the photo degradation rate after data imputation. Catboost + Adaboost with KNN imputation achieved the best performance for the original data. Ensemble learning and boosting methods (i.e., Catboost + Adaboost, XGB, and GBM) outperformed individual learning methods (i.e., ANN).

The strategy was further explored to augment the size of the original dataset with synthetically generated data using Vanilla GAN. The results indicated that incorporating generated data further increased the performance metrics for all ML models and mitigated overfitting problems with certain ML models. Once again, ensemble and boosting ML models demonstrated better performance.

SHAP analyses were utilized for the interpretability of ML models. The results showed that the photo-degradation rates of airborne contaminants were primarily affected by the dosage of photocatalyst, the reactor size, humidity, and the type of organic contaminant, followed by intensity, temperature, wavelength, and initial concentration. SHAP analyses further evaluated how the major contributing factors affect the ML model predictions, which are consistent with physics principles.

### Supplementary Information

Supplementary Information.

## Data Availability

All data generated or analysed during this study are included in this published article and its supplementary information files.
