# Supplementary material for: A novel interpretable machine learning model approach for the prediction of TiO2 photocatalytic degradation of air contaminants
Source: Sci Rep. 2024 Jun 6;14:13070. doi: 10.1038/s41598-024-62450-z (PMC11156991; doi:10.1038/s41598-024-62450-z)
Supplement: Supplementary file 1 — Supplementary Information. [file 41598_2024_62450_MOESM1_ESM.docx]

**SUPPLEMENTARY INFORMATION**

**Table S1** Summary of compiled data for this study.

| **No.** | **OC** | **I** | **W** | **D** | **H** | **T** | **R** | **Initial C** | **(-logK)** | **ref** |
| --- | --- | --- | --- | --- | --- | --- | --- | --- | --- | --- |
|  |  | **mW/cm2** | **nm** | **mg/cm2** | **%** | **⁰C** | **L** | **ppmv** | **min-1/cm2** |  |
| 1 | CCCCCCCC |  |  | 5.427 | 27.0 | 56.0 | 1.160 | 99.000 | 2.2867 | 27 |
| 2 | CCCCCCCCCC |  |  | 5.427 | 40.0 | 52.0 | 1.160 | 83.000 | 3.1819 | 27 |
| 3 | CCCCCC |  |  | 5.427 | 40.0 | 60.0 | 1.160 | 77.900 | 2.4209 | 27 |
| 4 | CC1CCCCC1 |  |  | 5.427 | 35.0 | 59.0 | 1.160 | 121.600 | 2.5692 | 27 |
| 5 | CC(C)CC(C)(C)C |  |  | 5.427 | 35.0 | 58.0 | 1.160 | 100.650 | 2.4490 | 27 |
| 6 | CCCCC | 21.00 |  | 0.012 | 500.0 | 85.0 | 0.145 | 5000.000 | 3.8329 | 28 |
| 7 | CCCCC | 21.00 |  | 0.012 | 20.0 | 85.0 | 0.145 | 5000.000 | 4.3100 | 28 |
| 8 | CCCCC | 21.00 |  | 0.012 | 300.0 | 85.0 | 0.145 | 5000.000 | 3.9298 | 28 |
| 9 | CCCCC | 21.00 |  | 0.012 | 1600.0 | 85.0 | 0.145 | 5000.000 | 3.7785 | 28 |
| 10 | CO | 21.00 |  | 0.012 | 20.0 | 85.0 | 0.145 | 5000.000 | 3.1516 | 28 |
| 11 | CO | 21.00 |  | 0.012 | 500.0 | 85.0 | 0.145 | 5000.000 | 3.0770 | 28 |
| 12 | CC(C)O | 21.00 |  | 0.012 | 20.0 | 85.0 | 0.145 | 5000.000 | 2.6351 | 28 |
| 13 | CC(C)O | 21.00 |  | 0.012 | 500.0 | 85.0 | 0.145 | 5000.000 | 3.1141 | 28 |
| 14 | Cc1ccccc1 | 21.00 |  | 0.012 | 20.0 | 85.0 | 0.145 | 5000.000 | 4.2700 | 28 |
| 15 | Cc1ccccc1 | 21.00 |  | 0.012 | 500.0 | 85.0 | 0.145 | 5000.000 | 4.0800 | 28 |
| 16 | ClCCl | 21.00 |  | 0.012 | 20.0 | 85.0 | 0.145 | 5000.000 | 4.4900 | 28 |
| 17 | ClCCl | 21.00 |  | 0.012 | 500.0 | 85.0 | 0.145 | 5000.000 | 4.4600 | 28 |
| 18 | c1ccncc1 | 21.00 |  | 0.012 | 20.0 | 85.0 | 0.145 | 5000.000 | 3.6472 | 28 |
| 19 | c1ccncc1 | 21.00 |  | 0.012 | 500.0 | 85.0 | 0.145 | 5000.000 | 3.6198 | 28 |
| 20 | c1ccccc1 | 1.55 | 325.0 |  | 50.0 | 30.0 | 1.920 |  | 4.1200 | 29 |
| 21 | Cc1ccccc1 | 1.55 | 325.0 |  | 50.0 | 30.0 | 1.920 |  | 3.9706 | 29 |
| 22 | c1ccccc1 | 1.60 | 300.0 | 96.000 | 40.6 | 46.3 | 0.676 | 6.484 | 3.0301 | 30 |
| 23 | c1ccccc1 | 4.00 | 300.0 | 96.000 | 41.8 | 45.7 | 0.676 | 17.640 | 2.9706 | 30 |
| 24 | Cc1ccccc1 | 1.60 | 300.0 | 96.000 | 41.3 | 45.6 | 0.676 | 30.290 | 3.0000 | 30 |
| 25 | Cc1ccccc1 | 4.00 | 300.0 | 96.000 | 38.6 | 44.3 | 0.676 | 14.740 | 2.8665 | 30 |
| 26 | Cc1ccc(C)cc1 | 1.60 | 300.0 | 96.000 | 37.6 | 46.6 | 0.676 | 10.060 | 2.6364 | 30 |
| 27 | Cc1ccc(C)cc1 | 4.00 | 300.0 | 96.000 | 41.1 | 42.4 | 0.676 | 16.460 | 2.5331 | 30 |
| 28 | CCCC | 28.00 | 366.0 |  | 100.0 | 25.0 | 0.275 | 873.000 | 3.4072 | 31 |
| 29 | CCCC | 28.00 | 366.0 |  | 100.0 | 25.0 | 0.275 | 291.000 | 3.2550 | 31 |
| 30 | CCCC | 28.00 | 366.0 |  | 100.0 | 25.0 | 0.275 | 182.000 | 3.2050 | 31 |
| 31 | CCCC | 28.00 | 366.0 |  | 100.0 | 25.0 | 0.275 | 109.000 | 3.0789 | 31 |
| 32 | CCCCC | 28.00 | 366.0 |  | 100.0 | 25.0 | 0.275 | 232.000 | 2.9626 | 31 |
| 33 | CCCCC | 28.00 | 366.0 |  | 100.0 | 25.0 | 0.275 | 309.000 | 2.9314 | 31 |
| 34 | CCCCC | 28.00 | 366.0 |  | 100.0 | 25.0 | 0.275 | 386.000 | 3.0242 | 31 |
| 35 | CCCCCC | 28.00 | 366.0 |  | 100.0 | 25.0 | 0.275 | 204.000 | 2.7476 | 31 |
| 36 | CCCCCCC | 28.00 | 366.0 |  | 100.0 | 25.0 | 0.275 | 303.000 | 2.6968 | 31 |
| 37 | CCCCCCC | 28.00 | 366.0 |  | 100.0 | 25.0 | 0.275 | 425.000 | 2.7501 | 31 |
| 38 | CCO | 28.00 | 366.0 |  | 100.0 | 25.0 | 0.275 | 1523.000 | 2.0824 | 31 |
| 39 | CCO | 28.00 | 366.0 |  | 100.0 | 25.0 | 0.275 | 1219.000 | 2.1364 | 31 |
| 40 | CCCO | 28.00 | 366.0 |  | 100.0 | 25.0 | 0.275 | 5944.000 | 2.2243 | 31 |
| 41 | CC(C)O | 28.00 | 366.0 |  | 100.0 | 25.0 | 0.275 | 1162.000 | 2.3427 | 31 |
| 42 | CCCCO | 28.00 | 366.0 |  | 100.0 | 25.0 | 0.275 | 1943.000 | 2.3140 | 31 |
| 43 | CCCCO | 28.00 | 366.0 |  | 100.0 | 25.0 | 0.275 | 971.000 | 2.2407 | 31 |
| 44 | CCC(C)O | 28.00 | 366.0 |  | 100.0 | 25.0 | 0.275 | 668.000 | 2.3799 | 31 |
| 45 | CCC(O)CC | 28.00 | 366.0 |  | 100.0 | 25.0 | 0.275 | 828.000 | 2.2407 | 31 |
| 46 | CCC(O)CC | 28.00 | 366.0 |  | 100.0 | 25.0 | 0.275 | 1657.000 | 2.3526 | 31 |
| 47 | CCCCCC |  |  | 6.809 |  | 65.0 | 1.160 | 100.000 | 2.6500 | 32 |
| 48 | C1CCCCC1 |  |  | 6.809 |  | 65.0 | 1.160 | 120.000 | 2.5563 | 32 |
| 49 | CCCCCCCC |  |  | 6.809 |  | 65.0 | 1.160 | 100.000 | 2.5627 | 32 |
| 50 | CC(C)CC(C)(C)C |  |  | 6.809 |  | 65.0 | 1.160 | 100.000 | 2.6745 | 32 |
| 51 | CC=O | 1.00 | 351.0 | 0.475 |  | 25.0 | 216.000 | 0.400 | 4.9000 | 33 |
| 52 | CC=O | 1.00 | 351.0 | 0.475 |  | 25.0 | 216.000 | 1.000 | 4.8600 | 33 |
| 53 | CC=O | 1.00 | 351.0 | 0.475 |  | 25.0 | 216.000 | 5.000 | 4.8600 | 33 |
| 54 | CSC | 1.00 | 351.0 | 0.475 |  | 25.0 | 216.000 | 0.030 | 4.1600 | 33 |
| 55 | CSSC | 1.00 | 351.0 | 0.475 |  | 25.0 | 216.000 | 0.030 | 4.3000 | 33 |
| 56 | CS | 1.00 | 351.0 | 0.475 |  | 25.0 | 4.300 | 0.020 | 4.3700 | 33 |
| 57 | Cc1ccccc1 | 10.00 | 365.0 |  | 50.0 | 23.0 | 120.000 | 0.052 | 2.3251 | 34 |
| 58 | Cc1ccccc1 | 10.00 | 365.0 |  | 50.0 | 23.0 | 120.000 | 0.097 | 2.3251 | 34 |
| 59 | Cc1ccccc1 | 10.00 | 365.0 |  | 50.0 | 23.0 | 120.000 | 0.260 | 2.3251 | 34 |
| 60 | CCCCCCCCCC | 10.00 | 365.0 |  | 50.0 | 23.0 | 120.000 | 0.052 | 2.3251 | 34 |
| 61 | CCCCCCCCCC | 10.00 | 365.0 |  | 50.0 | 23.0 | 120.000 | 0.095 | 2.3251 | 34 |
| 62 | CCCCCCCCCC | 10.00 | 365.0 |  | 50.0 | 23.0 | 120.000 | 0.284 | 2.3251 | 34 |
| 63 | C=O | 1.00 | 365.0 |  | 40.0 | 22.0 | 0.500 | 280.000 | 2.1612 | 35 |
| 64 | CO | 2.48 | 365.0 | 3.200 | 23.0 | 50.0 | 0.405 | 650.000 | 2.3571 | 36 |
| 65 | ClC=C(Cl)Cl | 2.48 | 365.0 | 3.200 | 23.0 | 50.0 | 0.405 | 538.000 | 2.4122 | 36 |
| 66 | CC(C)=O | 2.48 | 365.0 | 3.200 | 23.0 | 50.0 | 0.405 | 590.000 | 2.4382 | 36 |
| 67 | CCC(C)=O | 2.48 | 365.0 | 3.200 | 23.0 | 50.0 | 0.405 | 441.000 | 2.5166 | 36 |
| 68 | COCOC | 2.48 | 365.0 | 3.200 | 23.0 | 50.0 | 0.405 | 570.000 | 2.5040 | 36 |
| 69 | CC(C)CC(C)(C)C | 2.48 | 365.0 | 3.200 | 23.0 | 50.0 | 0.405 | 492.000 | 2.5203 | 36 |
| 70 | CC(C)C(C)=O | 2.48 | 365.0 | 3.200 | 23.0 | 50.0 | 0.405 | 455.000 | 2.8055 | 36 |
| 71 | CC(C)O | 2.48 | 365.0 | 3.200 | 23.0 | 50.0 | 0.405 | 750.000 | 2.8956 | 36 |
| 72 | COC(C)(C)C | 2.48 | 365.0 | 3.200 | 23.0 | 50.0 | 0.405 | 605.000 | 2.9333 | 36 |
| 73 | ClCCl | 2.48 | 365.0 | 3.200 | 23.0 | 50.0 | 0.405 | 398.000 | 2.9558 | 36 |
| 74 | ClC(Cl)Cl | 2.48 | 365.0 | 3.200 | 23.0 | 50.0 | 0.405 | 442.000 | 3.0889 | 36 |
| 75 | ClC(Cl)=C(Cl)Cl | 2.48 | 365.0 | 3.200 | 23.0 | 50.0 | 0.405 | 618.000 | 3.1466 | 36 |
| 76 | Cc1ccccc1 |  | 254.0 | 0.216 | 25.0 | 25.0 |  |  | 3.5169 | 37 |
| 77 | Cc1ccccc1 |  | 254.0 | 0.216 | 25.0 | 25.0 |  |  | 3.7005 | 37 |
| 78 | Cc1ccccc1 |  | 254.0 | 0.216 | 25.0 | 25.0 |  |  | 3.9970 | 37 |
| 79 | Cc1ccccc1 | 12.00 | 370.0 |  | 65.0 |  | 0.300 | 50.000 | 2.0189 | 38 |
| 80 | Clc1ccccc1Cl | 14.80 | 365.0 | 2.000 | 0.0 | 150.0 | 0.100 | 87.360 | 1.4295 | 39 |
| 81 | Clc1ccccc1Cl | 14.80 | 365.0 | 2.000 | 0.0 | 150.0 | 0.100 | 87.360 | 2.1079 | 39 |
| 82 | CCCCO | 0.65 | 565.0 | 0.167 | 85.0 |  | 1.720 | 580.000 | 4.7400 | 40 |
| 83 | ClC=C(Cl)Cl | 2.10 | 254.0 |  | 10.0 | 45.0 | 1.600 | 104.400 | 1.9787 | 41 |
| 84 | ClC=C(Cl)Cl | 2.10 | 254.0 |  | 10.0 | 45.0 | 1.600 | 182.700 | 2.1118 | 41 |
| 85 | ClC=C(Cl)Cl | 2.10 | 254.0 |  | 10.0 | 45.0 | 1.600 | 313.200 | 2.2702 | 41 |
| 86 | ClC=C(Cl)Cl | 2.10 | 254.0 |  | 10.0 | 45.0 | 1.600 | 365.400 | 2.3203 | 41 |
| 87 | ClC=C(Cl)Cl | 2.10 | 254.0 |  | 10.0 | 45.0 | 1.600 | 495.900 | 2.4249 | 41 |
| 88 | CC(C)=O | 2.10 | 254.0 |  | 0.0 | 45.0 | 1.600 | 78.300 | 2.4175 | 41 |
| 89 | CC(C)=O | 2.10 | 254.0 |  | 10.0 | 45.0 | 1.600 | 182.700 | 2.6532 | 41 |
| 90 | CC(C)=O | 2.10 | 254.0 |  | 10.0 | 45.0 | 1.600 | 313.200 | 2.8361 | 41 |
| 91 | CC(C)=O | 2.10 | 254.0 |  | 10.0 | 45.0 | 1.600 | 391.500 | 2.9175 | 41 |
| 92 | CC(C)=O | 2.10 | 254.0 |  | 0.0 | 45.0 | 1.600 | 495.900 | 3.0067 | 41 |
| 93 | CO | 2.10 | 254.0 |  | 10.0 | 45.0 | 1.600 | 156.600 | 2.6156 | 41 |
| 94 | CO | 2.10 | 254.0 |  | 10.0 | 45.0 | 1.600 | 234.900 | 2.7534 | 41 |
| 95 | CO | 2.10 | 254.0 |  | 10.0 | 45.0 | 1.600 | 287.100 | 2.8805 | 41 |
| 96 | CO | 2.10 | 254.0 |  | 10.0 | 45.0 | 1.600 | 443.700 | 2.9420 | 41 |
| 97 | CO | 2.10 | 254.0 |  | 0.0 | 45.0 | 1.600 | 495.900 | 3.0124 | 41 |
| 98 | Cc1ccccc1 | 2.10 | 254.0 |  | 21.0 | 45.0 | 1.600 | 52.200 | 3.0669 | 41 |
| 99 | Cc1ccccc1 | 2.10 | 254.0 |  | 10.0 | 45.0 | 1.600 | 78.300 | 3.1453 | 41 |
| 100 | Cc1ccccc1 | 2.10 | 254.0 |  | 10.0 | 45.0 | 1.600 | 104.400 | 3.2116 | 41 |
| 101 | Cc1ccccc1 | 2.10 | 254.0 |  | 10.0 | 45.0 | 1.600 | 130.500 | 3.2692 | 41 |
| 102 | Cc1ccccc1 | 2.10 | 254.0 |  | 0.0 | 45.0 | 1.600 | 156.600 | 3.3200 | 41 |
| 103 | c1ccccc1 |  | 254.0 | 3.500 | 0.0 |  | 0.040 | 3.300 | 2.2502 | 42 |
| 104 | c1ccccc1 |  | 254.0 | 3.500 | 19.8 |  | 0.040 | 3.300 | 2.1531 | 42 |
| 105 | c1ccccc1 |  | 254.0 | 3.500 | 32.4 |  | 0.040 | 3.300 | 2.0579 | 42 |
| 106 | c1ccccc1 |  | 254.0 | 3.500 | 58.6 |  | 0.040 | 3.300 | 1.9759 | 42 |
| 107 | c1ccccc1 |  | 254.0 | 3.500 | 114.4 |  | 0.040 | 3.300 | 1.9177 | 42 |
| 108 | Cc1ccccc1 |  | 254.0 | 3.500 | 0.0 |  | 0.040 | 9.500 | 2.3325 | 42 |
| 109 | Cc1ccccc1 |  | 254.0 | 3.500 | 19.8 |  | 0.040 | 9.500 | 2.0647 | 42 |
| 110 | Cc1ccccc1 |  | 254.0 | 3.500 | 34.5 |  | 0.040 | 9.500 | 1.9803 | 42 |
| 111 | Cc1ccccc1 |  | 254.0 | 3.500 | 55.3 |  | 0.040 | 9.500 | 1.9300 | 42 |
| 112 | Cc1ccccc1 |  | 254.0 | 3.500 | 66.0 |  | 0.040 | 9.500 | 1.9153 | 42 |
| 113 | Cc1ccccc1 |  | 254.0 | 3.500 | 83.5 |  | 0.040 | 9.500 | 1.8989 | 42 |
| 114 | CCc1ccccc1 |  | 254.0 | 3.500 | 0.0 |  | 0.040 | 9.500 | 2.0727 | 42 |
| 115 | CCc1ccccc1 |  | 254.0 | 3.500 | 13.4 |  | 0.040 | 9.500 | 1.8608 | 42 |
| 116 | CCc1ccccc1 |  | 254.0 | 3.500 | 41.0 |  | 0.040 | 9.500 | 1.7400 | 42 |
| 117 | CCc1ccccc1 |  | 254.0 | 3.500 | 63.8 |  | 0.040 | 9.500 | 1.7151 | 42 |
| 118 | CCc1ccccc1 |  | 254.0 | 3.500 | 93.7 |  | 0.040 | 9.500 | 1.7002 | 42 |
| 119 | Cc1cccc(C)c1 |  | 254.0 | 3.500 | 0.0 |  | 0.040 | 15.000 | 1.9177 | 42 |
| 120 | Cc1cccc(C)c1 |  | 254.0 | 3.500 | 19.6 |  | 0.040 | 15.000 | 1.9134 | 42 |
| 121 | Cc1cccc(C)c1 |  | 254.0 | 3.500 | 40.8 |  | 0.040 | 15.000 | 1.9145 | 42 |
| 122 | Cc1cccc(C)c1 |  | 254.0 | 3.500 | 64.4 |  | 0.040 | 15.000 | 1.9157 | 42 |
| 123 | Cc1cccc(C)c1 |  | 254.0 | 3.500 | 88.9 |  | 0.040 | 15.000 | 1.9170 | 42 |
| 124 | C1CO1 | 1.20 | 352.0 | 2.300 |  | 25.0 | 0.500 | 0.001 | 2.6628 | 43 |
| 125 | C1CO1 | 1.20 | 352.0 | 2.300 |  | 25.0 | 0.500 | 0.001 | 2.3203 | 43 |
| 126 | C1CO1 | 1.20 | 352.0 | 2.300 |  | 25.0 | 0.500 | 0.001 | 2.1065 | 43 |
| 127 | CC=O | 10.00 | 370.0 |  | 65.0 | 70.0 | 0.200 | 50.000 | 3.0608 | 44 |
| 128 | CC=O | 10.00 | 370.0 |  | 65.0 | 70.0 | 0.200 | 50.000 | 2.0926 | 44 |
| 129 | Cc1ccccc1 | 10.00 | 370.0 |  | 65.0 | 70.0 | 0.200 | 20.000 | 2.2602 | 44 |
| 130 | Cc1ccccc1 | 10.00 | 370.0 |  | 65.0 | 70.0 | 0.200 | 20.000 | 2.3140 | 44 |
| 131 | Cc1ccccc1 | 10.00 | 370.0 |  | 65.0 | 70.0 | 0.200 | 20.000 | 2.3851 | 44 |
| 132 | Cc1ccccc1 | 10.00 | 370.0 |  | 65.0 | 70.0 | 0.200 | 20.000 | 2.4771 | 44 |
| 133 | Cc1ccccc1 | 10.00 | 370.0 |  | 65.0 | 70.0 | 0.200 | 20.000 | 3.3739 | 44 |
| 134 | Cc1ccccc1 | 10.00 | 370.0 |  | 65.0 | 70.0 | 0.200 | 20.000 | 3.9622 | 44 |
| 135 | Cc1ccccc1 | 10.00 | 370.0 |  | 65.0 | 70.0 | 0.200 | 20.000 | 2.3040 | 44 |
| 136 | Cc1ccccc1 | 10.00 | 370.0 |  | 65.0 | 70.0 | 0.200 | 20.000 | 2.1280 | 44 |
| 137 | Cc1ccccc1 | 10.00 | 370.0 |  | 65.0 | 70.0 | 0.200 | 20.000 | 2.4094 | 44 |
| 138 | Cc1ccccc1 | 10.00 | 370.0 |  | 65.0 | 70.0 | 0.200 | 20.000 | 3.5139 | 44 |
| 139 | Cc1ccccc1 |  | 254.0 | 0.013 | 65.0 | 350.0 | 1.500 | 1.000 | 3.3739 | 45 |
| 140 | Cc1ccccc1 |  | 254.0 | 0.013 | 65.0 | 350.0 | 1.500 | 1.000 | 3.9622 | 45 |
| 141 | Cc1ccccc1 | 5.80 | 250.0 |  | 35.0 | 25.0 |  | 0.009 | 2.3040 | 46 |
| 142 | CC(C)=O | 5.80 | 250.0 |  | 35.0 | 25.0 |  | 0.006 | 2.1280 | 46 |
| 143 | Cc1cccc(C)c1 | 5.80 | 250.0 |  | 35.0 | 25.0 |  | 0.011 | 2.4094 | 46 |
| 144 | Cc1ccccc1 | 3.64 | 365.0 | 0.065 |  | 25.0 | 2.000 | 49.000 | 3.5139 | 47 |
| 145 | Cc1ccccc1 | 3.64 | 365.0 | 0.065 |  | 25.0 | 2.000 | 49.000 | 3.4284 | 47 |
| 146 | Cc1ccccc1 | 3.64 | 365.0 | 0.065 |  | 25.0 | 2.000 | 49.000 | 3.1871 | 47 |
| 147 | C=O | 30.00 | 358.0 | 0.400 | 40.0 |  | 5.000 | 0.001 | 3.3175 | 48 |
| 148 | C1CO1 | 30.00 | 358.0 | 0.400 | 40.0 |  | 5.000 | 0.007 | 3.9484 | 48 |
| 149 | COC#C | 30.00 | 358.0 | 0.400 | 40.0 |  | 5.000 | 0.004 | 3.9484 | 48 |
| 150 | C1OCC=C1 | 30.00 | 358.0 | 0.400 | 40.0 |  | 5.000 | 0.004 | 3.9484 | 48 |
| 151 | Clc1ccccc1 | 30.00 | 358.0 | 0.400 | 40.0 |  | 5.000 |  | 3.9484 | 48 |
| 152 | Clc1ccccc1Cl | 30.00 | 358.0 | 0.400 | 40.0 |  | 5.000 |  | 3.9484 | 48 |
| 153 | Clc1ccc(Cl)c(Cl)c1 | 30.00 | 358.0 | 0.400 | 40.0 |  | 5.000 |  | 3.9484 | 48 |
| 154 | c1ccccc1 | 30.00 | 358.0 | 0.400 | 40.0 |  | 5.000 | 0.020 | 3.9484 | 48 |
| 155 | Cc1ccccc1 | 30.00 | 358.0 | 0.400 | 40.0 |  | 5.000 | 0.400 | 3.9484 | 48 |
| 156 | Cc1cccc(C)c1 | 30.00 | 358.0 | 0.400 | 40.0 |  | 5.000 |  | 3.9484 | 48 |
| 157 | CC(C)=C1CCC(=CC1)C | 30.00 | 358.0 | 0.400 | 40.0 |  | 5.000 |  | 5.1200 | 48 |
| 158 | CC(C)C#C | 30.00 | 358.0 | 0.400 | 40.0 |  | 5.000 |  | 5.0000 | 48 |
| 159 | CCC(C)=O | 30.00 | 358.0 | 0.400 | 40.0 |  | 5.000 |  | 4.4600 | 48 |
| 160 | CO | 30.00 | 358.0 | 0.400 | 40.0 |  | 5.000 |  | 4.5200 | 48 |
| 161 | C(C#N)([2H])([2H])[2H] | 30.00 | 358.0 | 0.400 | 40.0 |  | 5.000 |  | 4.6500 | 48 |
| 162 | CCO | 30.00 | 358.0 | 0.400 | 40.0 |  | 5.000 |  | 4.7000 | 48 |
| 163 | C=O | 30.00 | 358.0 | 0.400 | 40.0 |  | 5.000 | 0.001 | 4.7000 | 48 |
| 164 | C1CO1 | 30.00 | 358.0 | 0.400 | 40.0 |  | 5.000 | 0.007 | 5.1200 | 48 |
| 165 | COC#C | 30.00 | 358.0 | 0.400 | 40.0 |  | 5.000 | 0.004 | 4.5600 | 48 |
| 166 | C1OCC=C1 | 30.00 | 358.0 | 0.400 | 40.0 |  | 5.000 | 0.004 | 4.4900 | 48 |
| 167 | Clc1ccccc1 | 30.00 | 358.0 | 0.400 | 40.0 |  | 5.000 | 0.002 | 4.3000 | 48 |
| 168 | Clc1ccccc1Cl | 30.00 | 358.0 | 0.400 | 40.0 |  | 5.000 | 0.001 | 4.6000 | 48 |
| 169 | Clc1ccc(Cl)c(Cl)c1 | 30.00 | 358.0 | 0.400 | 40.0 |  | 5.000 | 0.001 | 3.3175 | 48 |
| 170 | c1ccccc1 | 30.00 | 358.0 | 0.400 | 40.0 |  | 5.000 | 0.004 | 3.9484 | 48 |
| 171 | Cc1ccccc1 | 30.00 | 358.0 | 0.400 | 40.0 |  | 5.000 | 0.003 | 3.9484 | 48 |
| 172 | Cc1cccc(C)c1 | 30.00 | 358.0 | 0.400 | 40.0 |  | 5.000 | 0.003 | 3.9484 | 48 |
| 173 | CC(C)=C1CCC(=CC1)C | 30.00 | 358.0 | 0.400 | 40.0 |  | 5.000 | 0.001 | 3.9783 | 48 |
| 174 | CC(C)C#C | 30.00 | 358.0 | 0.400 | 40.0 |  | 5.000 | 0.002 | 3.9484 | 48 |
| 175 | CCC(C)=O | 30.00 | 358.0 | 0.400 | 40.0 |  | 5.000 |  | 3.9484 | 48 |
| 176 | CO | 30.00 | 358.0 | 0.400 | 40.0 |  | 5.000 |  | 3.9484 | 48 |
| 177 | C(C#N)([2H])([2H])[2H] | 30.00 | 358.0 | 0.400 | 40.0 |  | 5.000 |  | 3.9484 | 48 |
| 178 | CCO | 30.00 | 358.0 | 0.400 | 40.0 |  | 5.000 |  | 3.9484 | 48 |
| 179 | ClC=C(Cl)Cl | 0.36 | 253.7 | 0.300 |  | 25.0 | 3.300 | 0.380 | 2.0904 | 49 |
| 180 | ClC(Cl)Cl | 0.36 | 253.7 | 0.300 |  | 25.0 | 3.300 | 0.420 | 2.6498 | 49 |
| 181 | ClCCl | 0.36 | 253.7 | 0.300 |  | 25.0 | 3.300 | 0.520 | 3.1675 | 49 |
| 182 | Cc1ccccc1 | 0.36 | 253.7 | 0.300 |  | 25.0 | 3.300 | 0.290 | 3.0555 | 49 |
| 183 | c1ccccc1 | 0.36 | 253.7 | 0.300 |  | 25.0 | 3.300 | 0.400 | 3.5784 | 49 |
| 184 | ClC(Cl)(Cl)Cl | 0.36 | 253.7 | 0.300 |  | 25.0 | 3.300 | 0.360 | 0.0000 | 49 |
| 185 | c1ccccc1 | 80.00 | 0.0 | 0.100 | 43.0 | 25.0 |  | 20.000 | 4.3600 | 50 |
| 186 | Cc1ccccc1 | 80.00 | 0.0 | 0.100 | 43.0 | 25.0 |  | 20.000 | 4.0200 | 50 |
| 187 | Cc1cccc(C)c1 | 80.00 | 0.0 | 0.100 | 43.0 | 25.0 |  | 20.000 | 4.9500 | 50 |
| 188 | CCC(C)=O | 3.60 | 254.0 | 0.490 | 0.0 | 25.0 | 6.300 | 0.003 | 5.2800 | 51 |
| 189 | CCC(C)=O | 3.60 | 254.0 | 0.490 | 20.0 | 25.0 | 6.300 | 0.003 | 5.1300 | 51 |
| 190 | CCC(C)=O | 3.60 | 254.0 | 0.490 | 40.0 | 25.0 | 6.300 | 0.003 | 5.1100 | 51 |
| 191 | CCC(C)=O | 3.60 | 254.0 | 0.490 | 60.0 | 25.0 | 6.300 | 0.003 | 5.0600 | 51 |
| 192 | CCC(C)=O | 3.60 | 254.0 | 0.490 | 0.0 | 25.0 | 6.300 | 0.003 | 5.0900 | 51 |
| 193 | CCC(C)=O | 3.60 | 254.0 | 0.490 | 20.0 | 25.0 | 6.300 | 0.003 | 5.0600 | 51 |
| 194 | CCC(C)=O | 3.60 | 254.0 | 0.490 | 40.0 | 25.0 | 6.300 | 0.003 | 4.8700 | 51 |
| 195 | CCC(C)=O | 3.60 | 254.0 | 0.490 | 60.0 | 25.0 | 6.300 | 0.003 | 4.8800 | 51 |
| 196 | CC(C)O | 1.00 | 358.0 | 0.017 |  | 26.9 | 0.890 | 6.394 | 9.4600 | 52 |
| 197 | CC(C)O | 1.00 | 358.0 | 0.017 |  | 26.9 | 0.890 | 6.394 | 9.4300 | 52 |
| 198 | CC(C)O | 1.00 | 358.0 | 0.017 |  | 26.9 | 0.890 | 6.394 | 9.5400 | 52 |
| 199 | CC(C)O | 1.00 | 358.0 | 0.017 |  | 26.9 | 0.890 | 6.394 | 9.8000 | 52 |
| 200 | CC(C)O | 1.00 | 358.0 | 0.017 |  | 26.9 | 0.890 | 6.394 | 9.3600 | 52 |

**Overview of employed ML models**

***Artificial neural network (ANN) model***

The prediction and learning of the relationship between eight input variables, i.e., Smile, Intensity, Wavelength, Dosage, Humidity, temperature, Reactor, Initial concentration, and the reaction rate is the output were done by building an ANN model. ANNs are parallel architectures that can tackle challenging tasks. with an interconnection between computing elements (or artificial neurons) [74]. It has been widely used in curve fitting issues and is one of the main branches of artificial intelligence (AI) [75]. Furthermore, there are many processing components, such as neurons, and the layers are connected to one another by weights, just like in genuine nervous systems. [76]. The common architecture of an ANN model is presented in **Fig. S1** Basically, an artificial neuron can be separated into five divisions: inputs, weights, sum function, activation function, and outputs. The input layer receives data from the external environment, and the neurons in the hidden and output layers process this information using weights and a sum function. The sum function calculates the net input of a neuron based on its inputs and weights [77]. Then, the neuron applies a non-linear activation function to the net input to produce an output, which is sent to the following neurons. The process of a neuron involves both linear and non-linear mathematical operations, which are represented by equations (4) and (5).

${net}_{j}=\sum_{i=1}^{n} In_{i}w_{ij}+b_{j}$ (4)

${Out}_{j}={f(net}_{j})$ (5)

where $In_{i}$ and $b_{j}$ are the $i$th input signal and the bias value of $j$th neuron, respectively, $w_{ij}$ is the linking weight between $i$th input signal and $j$th neuron and f is the activation function. Dropout is a technique used during the training phase to randomly avoid some neurons in the hidden layer, which supports preventing overfitting of the training set and produces more robust models [78]. The optimal number of layers and neurons in the hidden layers are obtained through a trial-and-error approach. The transfer function adopted was rectified linear unit (ReLU), is a commonly used activation function between the hidden layers [79]. The Adam training function with a tunable learning rate and a batch size of 64 was used for optimization over 500 cycles. The 10-fold cross-validation method was used to train and test the ANN model, with one subgroup always reserved for validation, and the results were averaged from each of the ten iterations.

**
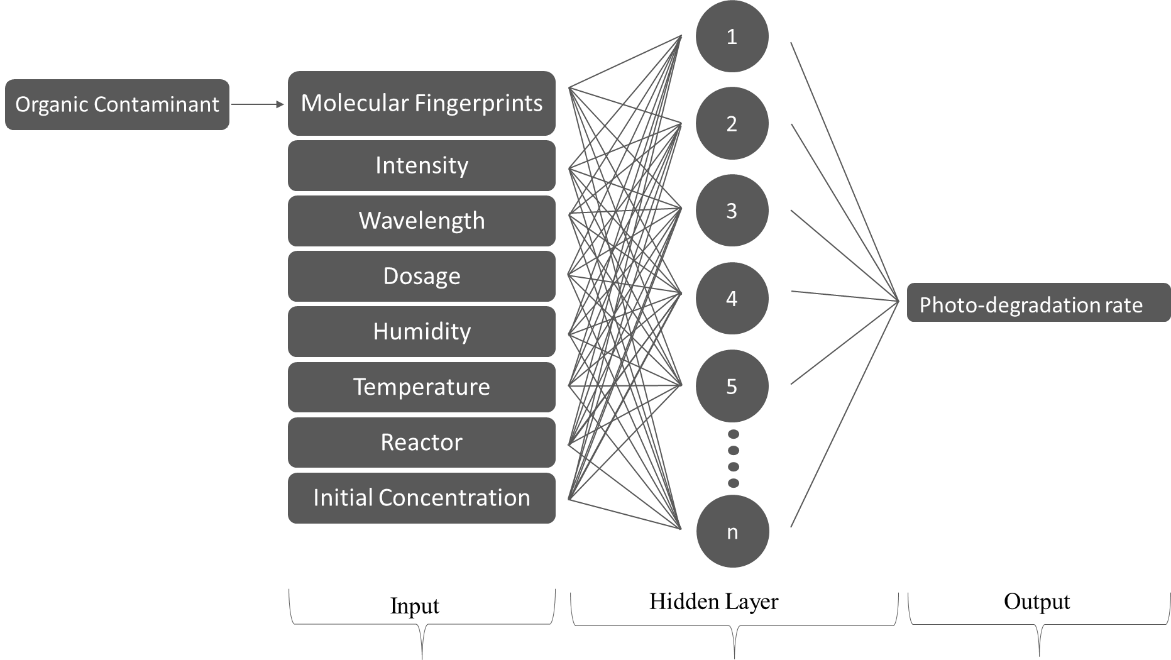
**

**Fig. S1** Architecture of artificial neural network.

***Gradient booster regressor (GBR)***

To reduce the error in the predictions and increase the efficiency of the model, the gradient boosting regressor (GBR) model [80] has been adopted. The GBR was designed by Friedman as a way to extend the boosting algorithm to regression problems. Three elements are part of the GBR structure, which is regarded as a generalization of gradient boosting: a loss function that must be minimized; a weak learner used to make predictions; and an additive model that multiplies weak learners [81]. The key concept of this algorithm involves developing new base learners that are most highly correlated with the negative gradient of the loss function, which in turn is correlated with the full ensemble [81]. The GBR algorithm was implemented using the Python package Scikit-Optimize, combined with Bayesian Optimization to select the best hyperparameters. We choose to use scikit-optimize[82] whose method BayesSearchCV has a very well-implemented PYTHON interface compatible with sci-kit learn. The GBR model was trained and tested using the 10-fold cross-validation method, where one subgroup was consistently reserved for validation. The results were averaged across the ten iterations.

***Extreme Gradient Boosted Decision Trees (XGBoost)***

Extreme Gradient Boosting (XGBoost) a type of supervised algorithm, was created by Chen and Guestrin [83] and has become widely used and popular. By using a more regularized model generation, the improved gradient boosting machine known as XGBoost can manage over-fitting more successfully [83]. Furthermore, even with many different data types, it is well known that XGBoost is able to produce good solutions than other ML algorithms due to its rapidity, efficiency, and scalability [84,85].

XGBoost utilizes K additive function to predict the output, which is characterized as equation (6).

$\hat{y}_{o}\sum_{k=1}^{K} f_{k}\left( x_{i} \right), f_{k}\in F$ (6)

In the equation (3), K corresponds to the number of regression trees in a tree ensemble, $f$ is a function in functional space (F), is a function in the functional space (F), and F represents the collection of all possible Classification and Regression Trees (CARTs) available. Additionally, $x_{i}$ refers to the input feature vector for the $i^{th}$data point a given dataset $D=\left\{ x_{i},y_{i} \right\}$, where $i$ ranges from 1 to n (with n being the total number of data points).

The gradient boosting technique is particularly useful for identifying the optimal tree model by utilizing more precise estimates. By sequentially learning a series of weak learners, the model uses a sequential training technique to develop a strong learner. Every step uses the gradient descent optimization algorithm to train a weak learner to minimize the loss function [86]. In addition, it applies first and second-order gradient statistics to improve the loss function. Additional advantages of XGBoost is related to the simplicity to parallelize and distribute training across clusters [87]. The XGB model underwent training and testing via the 10-fold cross-validation method, with one subgroup consistently set aside for validation. The results were then averaged across the ten iterations.

***CatBoost***

The CatBoost algorithm is a supervised machine learning approach that can be applied for regression, classification, and time series analysis. It is a machine learning framework that utilizes gradient boosting trees and can handle categorical features and string-type features [88].  It is a gradient-boosting library, and it has both the GPU and CPU implementation learning algorithms, which make it faster than other gradient-boosting libraries. In addition, gradient boosting is a powerful machine learning approach, which shows great potential in learning problems with heterogeneous features, noisy data, and data of complex dependencies. Additionally, catboost adopted a novel method for calculating the leaf values while choosing the network structure, which lessens the likelihood of overfitting [89]. The Catboost model was trained and tested using the 10-fold cross-validation method, with one subgroup always reserved for validation. The average results were computed across the ten iterations.

**Hyperparameter Optimization**

***Hyperparameter tuning using Bayesian Optimization***

Bayesian optimization is a probabilistic technique used to minimize functions, and it has been applied to hyperparameter tuning in machine learning problems [90]. Previous studies have shown that Bayesian optimization is effective for ANN and GBR, which is why it has been adopted for these models [91,92]. BO includes several elements, such as evaluating the loss on the verification set when applying a set of hyperparameters in machine learning, defining the search space that contains the range of hyperparameter values to be explored, using an optimization algorithm to develop the objective function and select hyperparameter values for evaluation, and considering the evaluation performance of the objective function along with hyperparameter values and losses on the validation set when generating optimization results [93].

***Hyperparameter tuning using Hyperopt***

Hyperparameter optimization refers to the task of optimizing a particular mapping function across a configuration space that has a graph-like structure [94]. Three algorithms for Hyperopt are used. The first is random search [95] which is an ancestral sampling of the search space, expressed in terms of directed graphical models. The annealing algorithm [95] is another approach for hyperparameter optimization that outperforms random search in this regard, and it can quickly optimize a gradient-free function over a hyperopt-style space. At the beginning, the algorithm selects points randomly, but as time passes, the distributions become more focused around the best performing point. The third algorithm, known as the Tree of Parzen Estimators (TPE), is also used [95]. The underlying assumptions of the algorithm are linked to specific modeling assumptions, in which maximizing Expected Improvement (EI) can be connected to the maximization of the ratio $\frac{P(X|y<\tau)}{P(X|y\geq\tau)}$ [96]. The TPE algorithm models densities over an N-dimensional search space without conditional parameters, using two product-of-marginal [Parzen] estimators that have the same form. The model of points from a dataset $D$ with $y<\tau$ is expressed as shown in equation (7).

$Q\left( X | y<\tau\right)= \prod_{i} \sum_{(x,y)\epsilon D|y<\tau} \frac{1}{\sqrt{2\pi\sigma_{ij}}}e^{{\frac{(x_{ij}-x_{i)}}{{2\sigma}_{ij}^{2}}}^{2}}$ (7)

**Ensemble Catboost + Adaboost**

In supervised machine learning problems, ensemble learning is a broad name for techniques that mix numerous models to create predictions. Its ability to combine multiple models, make it stand out, as the error of a single model is improved by other models which therefore makes the result performance better than a single model In addition, it also helps to reduce the possibility of overfitting because, when a dataset is limited, a learning algorithm is opened to provide a large number of hypotheses that accurately predict all of the training samples and produces inaccurate predictions for unobserved data. Therefore, getting the average of different hypotheses reduces the chances of selecting an incorrect hypothesis which caused the accuracy to be improved. For this write-up, we combined AdaBoost and CatBoost together, as AdaBoost is a popular dependent algorithm for creating ensemble models which focused on the result that was previously misclassified when training with the new model [97]. Furthermore, the model works by using multiples iteration, and in each, the weight of the result that is misclassified is reduced, while the weight of the correctly classified is increased. In addition, the weight of the base learner used in ensemble learning is also assigned based on their performance [98].

**Performance of ML model with removal of samples with missing data**

Without the use of imputation methods, the dataset would have been limited to a maximum of 28 complete samples out of the 200 samples with incomplete data. Even with the removal of some inputs, this would not have provided enough data for obtaining accurate results. The use of only 28 samples for modeling would have yielded poor results, as demonstrated in **Table *S2.*** The R² values for the training sets were low, sometimes even negative, and the R² values for the testing sets were all negative, indicating inconsistency. The low quantity of samples, especially in the testing set, was the reason for these poor results. The MSE values were also extremely low, which is usually desirable, but in this case, the values were close to zero and not reliable due to the limited quantity of samples. The imputation methods allowed for the use of all available data and thus produced much more accurate results.

**Table *S2* -** Performance comparison for prediction of photo-degradation rate of air contaminants without imputation method from various ML Techniques.

|  |  | **R²** | | **MSE** | |
| --- | --- | --- | --- | --- | --- |
| **Model** | HYPEROPT method for XGB | **Train** | **Test** | **Train** | **Test** |
| ANN with BO |  | 0.566 | -7.332 | 0.000029 | 0.000010 |
| Catboost + adaboost |  | 0.662 | -2.279 | 0.000020 | 0.000004 |
| GBM with BO |  | 0.653 | -4.938 | 0.000023 | 0.000007 |
| XGB with HYPEROPT | Random search | -0.020 | -8.284 | 0.000053 | 0.000002 |
|  | Annealing | -0.001 | -27.803 | 0.000052 | 0.000008 |
|  | TPE | -0.003 | -31.938 | 0.000052 | 0.000009 |


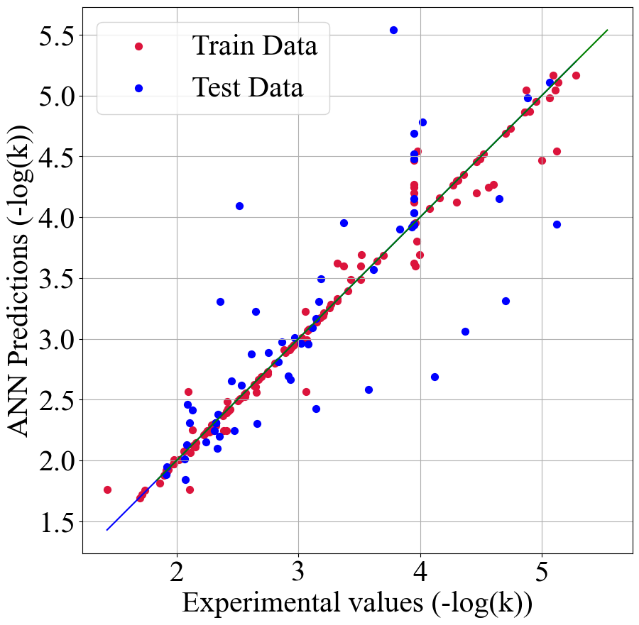

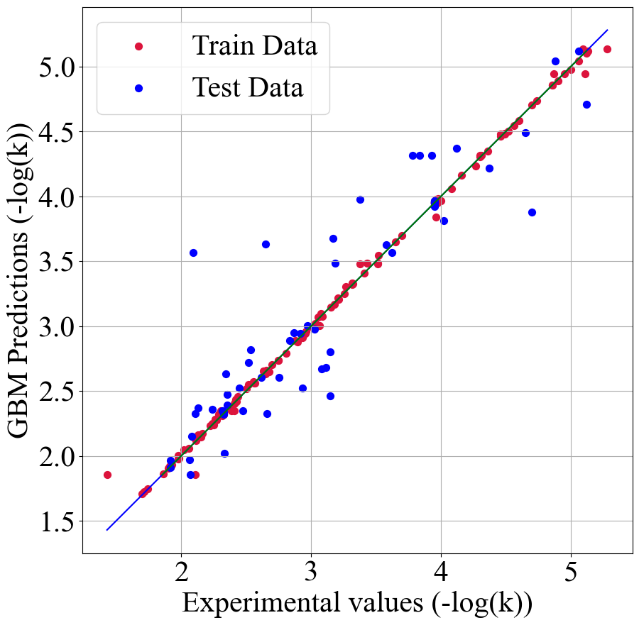


(a) (b)


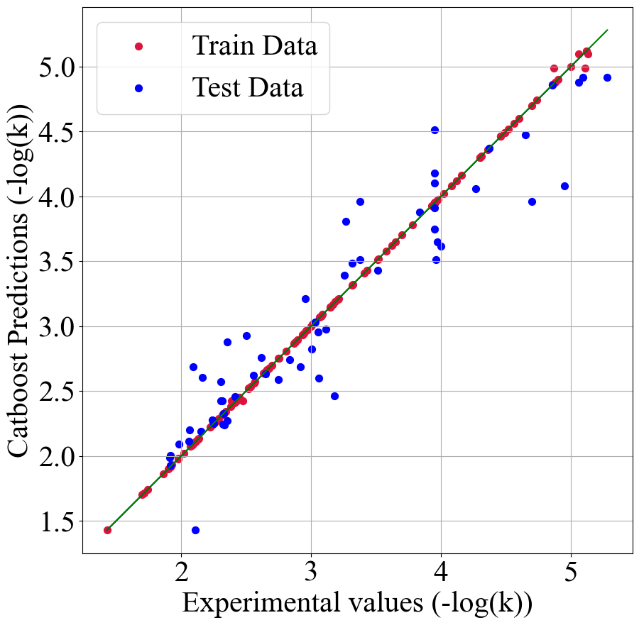

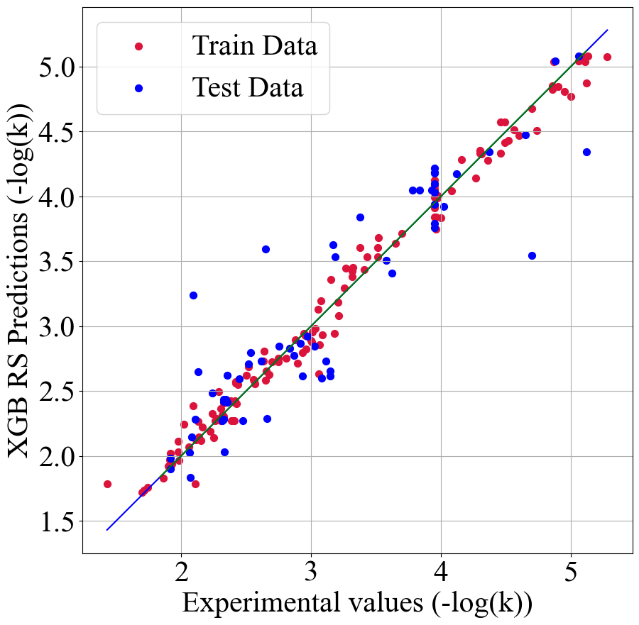


(c) (d)


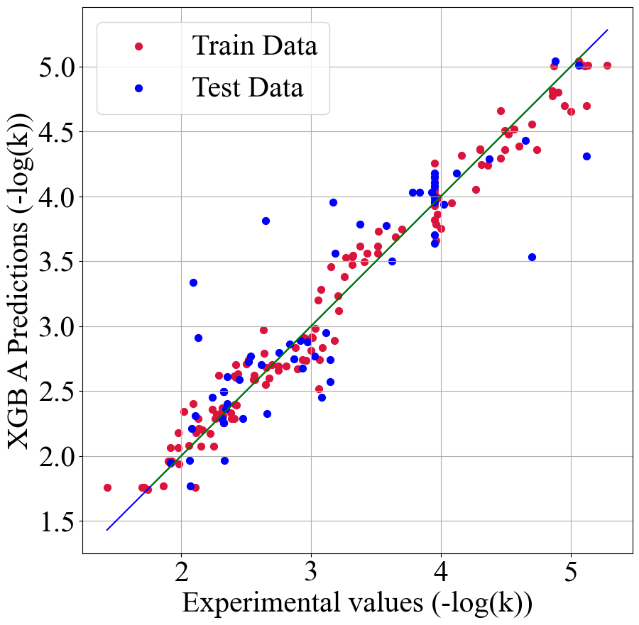

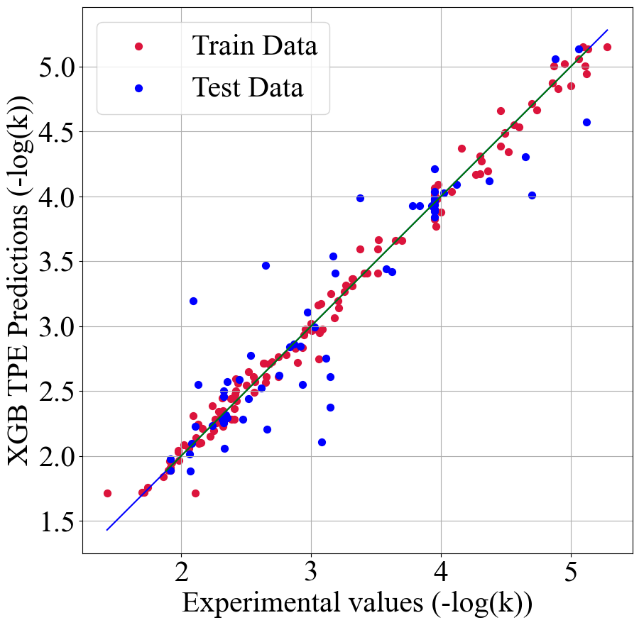


(e) (f)

**Fig. S2** The scatter plots of the predicted vs. experimental photo-degradation rate constants –log(k) using MF imputation for (a) ANN, (b) Catboost + adaboost, (c) GBM, (d) XGB Random search, (e) XGB Annealing, and (f) XGB TPE.


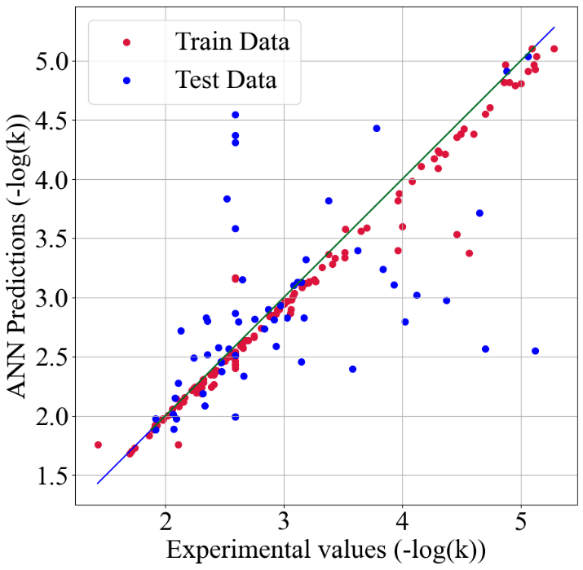

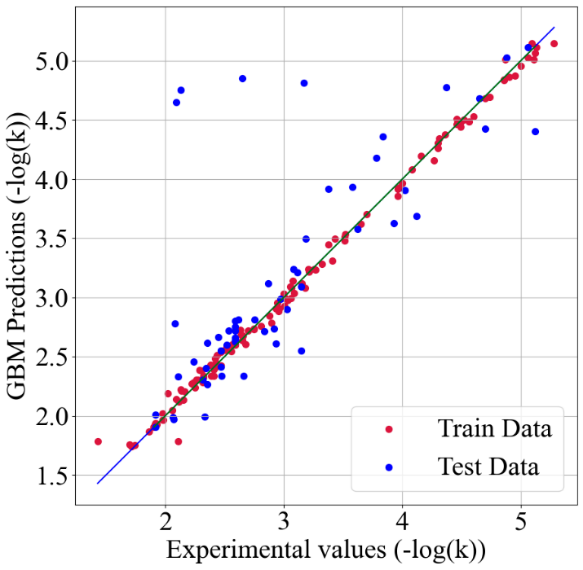


(a) (b)


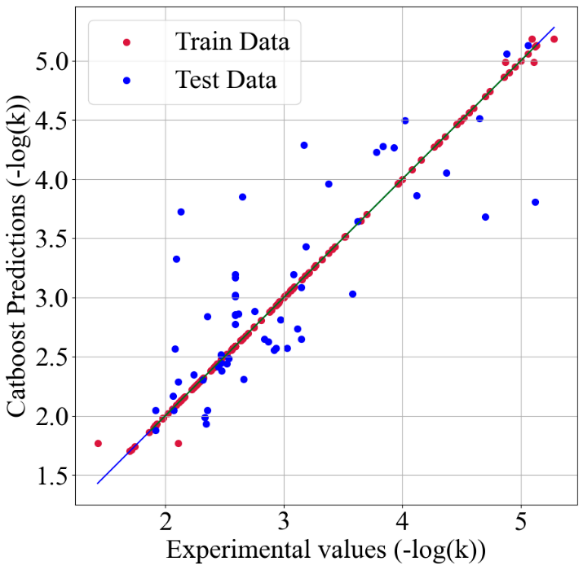

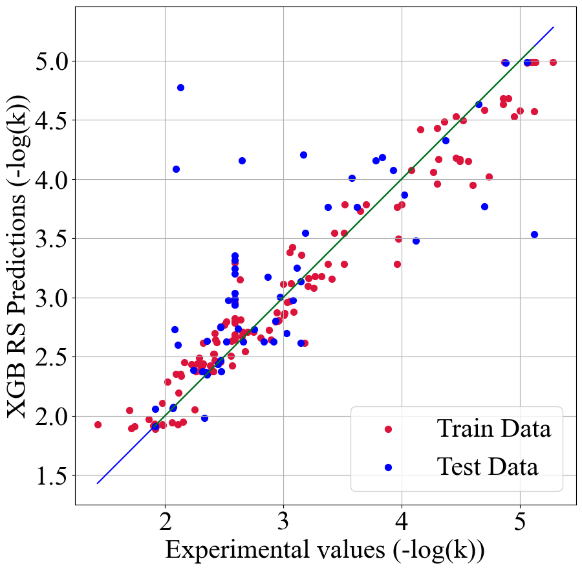


(c) (d)


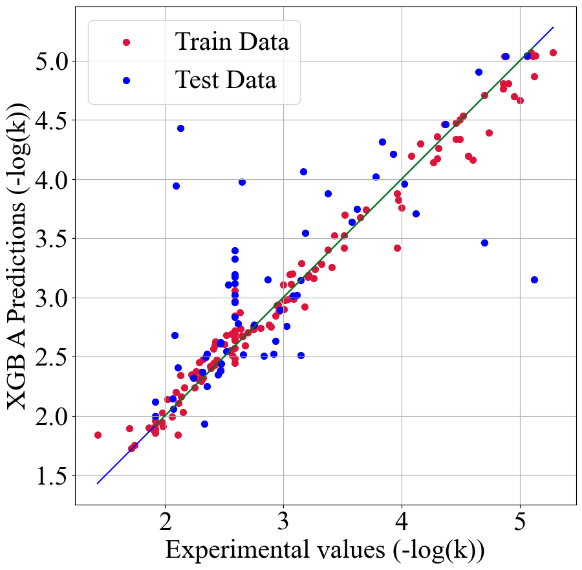

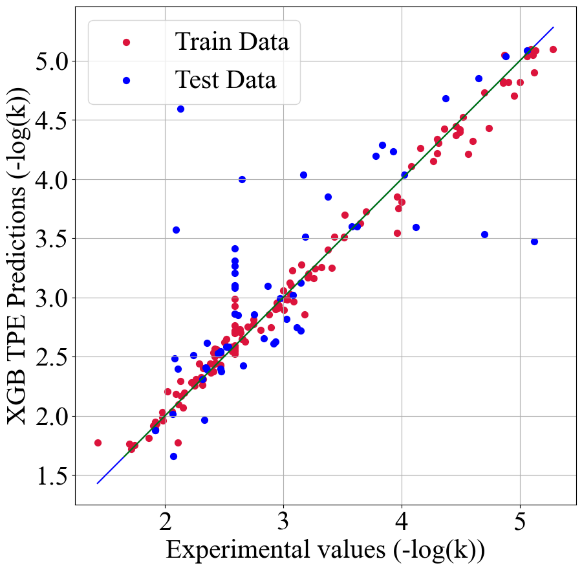


(e) (f)

**Fig. S3** The scatter plots of the predicted vs. experimental photo-degradation rate constants –log(k) using Datawig imputation for (a) ANN, (b) Catboost + adaboost, (c) GBM, (d) XGB Random search, (e) XGB Annealing, and (f) XGB TPE.


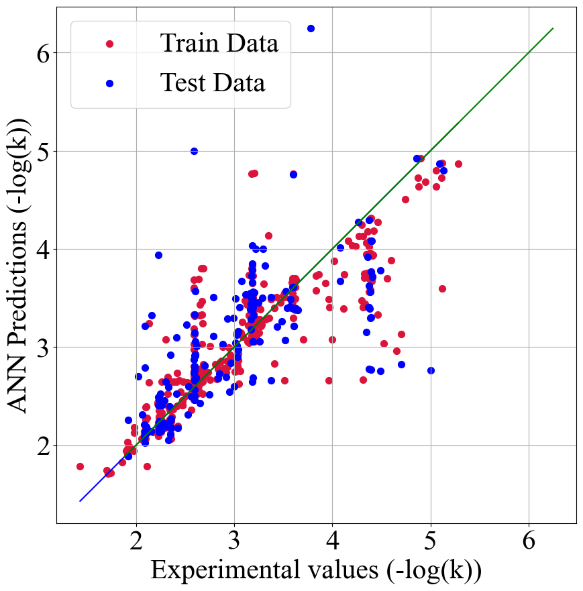

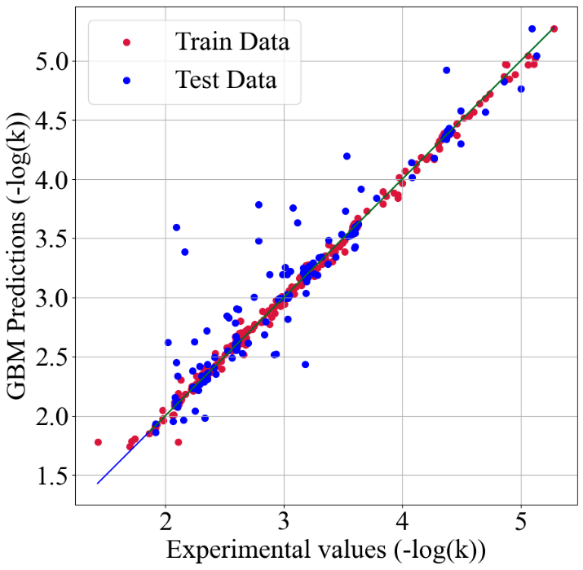


(a) (b)


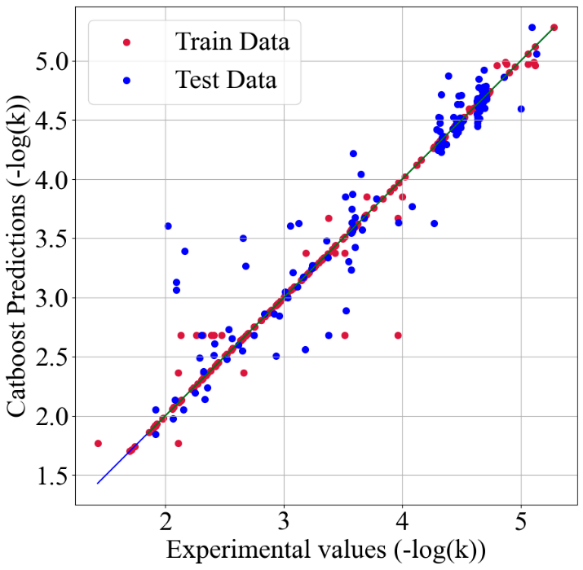

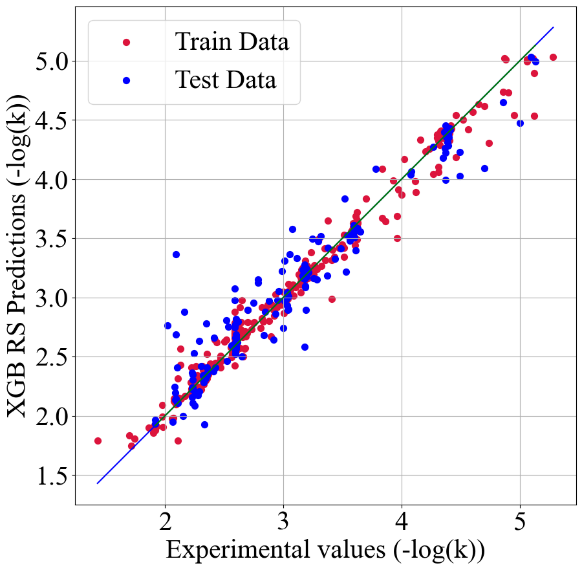


(c) (d)


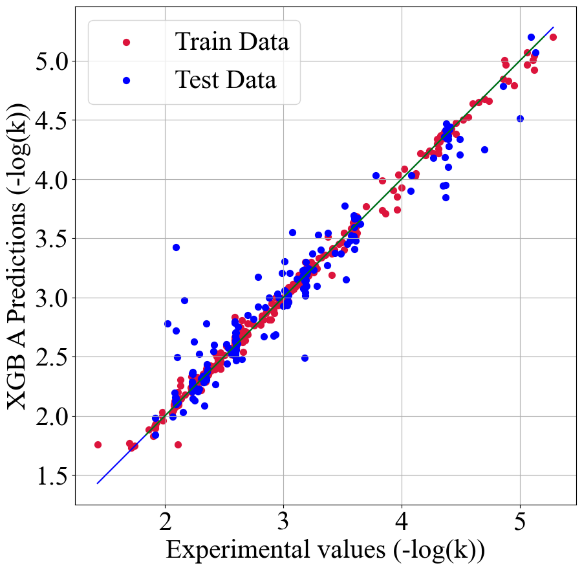

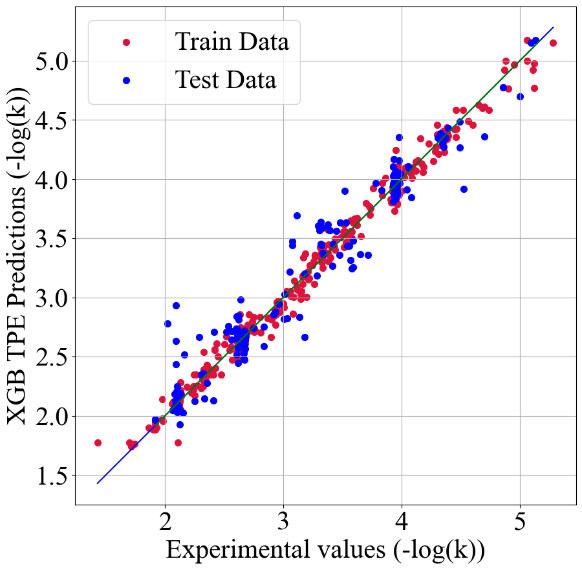


(e) (f)

**Fig. S4** The scatter plots of the predicted vs. experimental photo-degradation rate constants –log(k) using Datawig imputation AND synthetic data for (a) ANN, (b) Catboost + adaboost, (c) GBM, (d) XGB Random search, (e) XGB Annealing, and (f) XGB TPE.


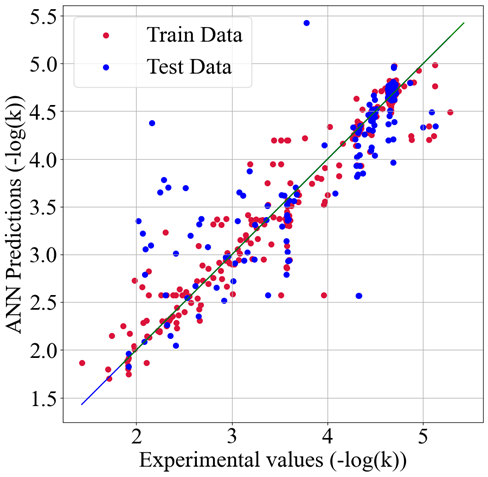

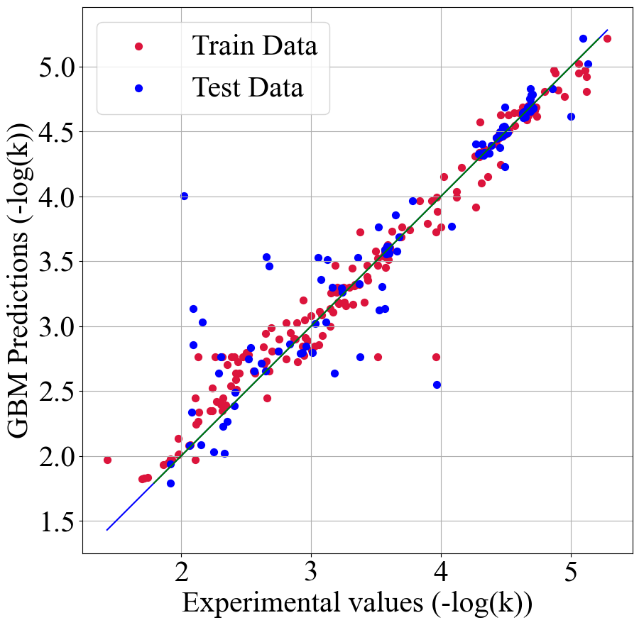


(a) (b)


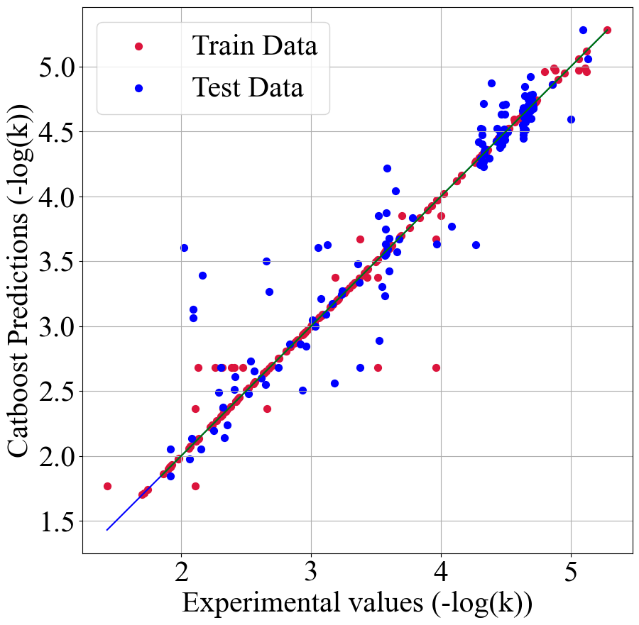

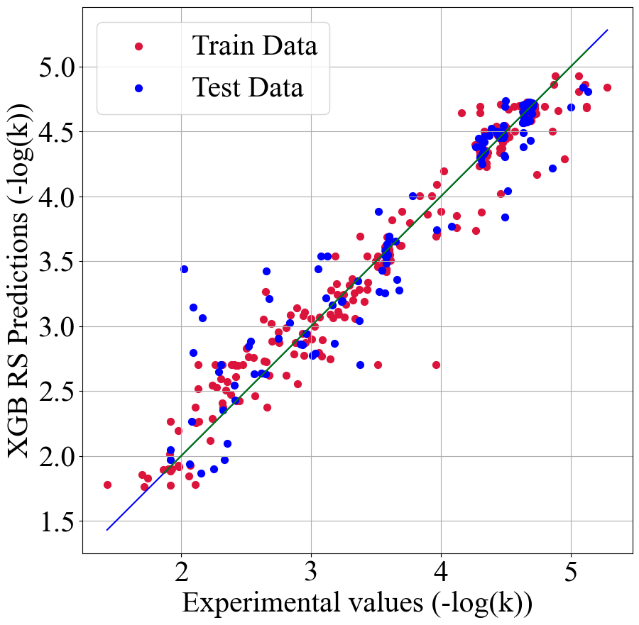


(c) (d)


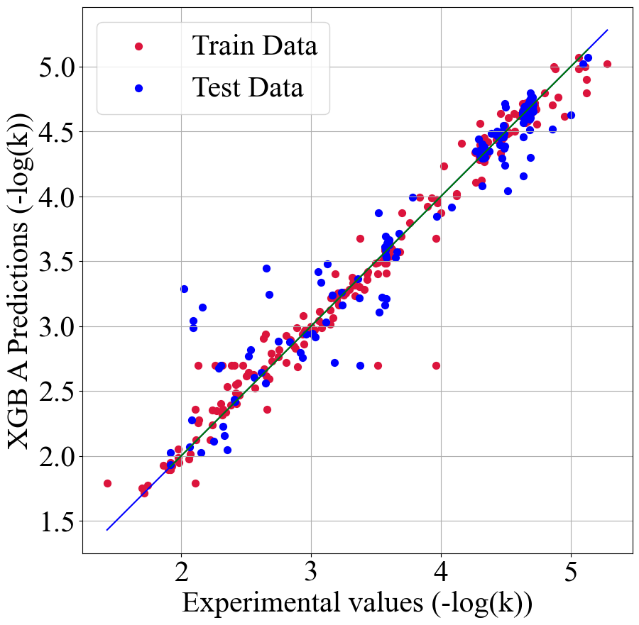

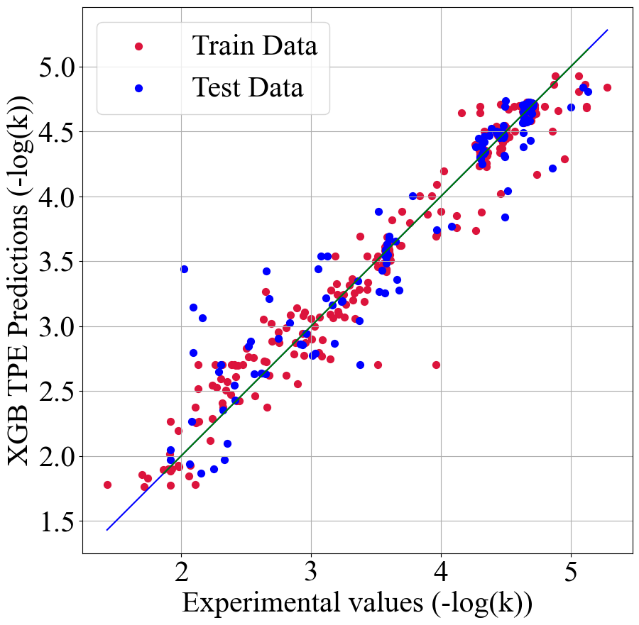


(e) (f)

**Fig. S5** The scatter plots of the predicted vs. experimental photo-degradation rate constants –log(k) using KNN imputation AND synthetic data for (a) ANN, (b) Catboost + adaboost, (c) GBM, (d) XGB Random search, (e) XGB Annealing, and (f) XGB TPE.

Supplementary Materials Reference

[74] M. Pala, E. Özbay, A. Öztaş, M.I. Yuce, Appraisal of long-term effects of fly ash and silica fume on compressive strength of concrete by neural networks, Constr. Build. Mater. 21 (2007) 384–394. https://doi.org/10.1016/j.conbuildmat.2005.08.009.

[75] E.M. Golafshani, A. Behnood, M. Arashpour, Predicting the compressive strength of normal and High-Performance Concretes using ANN and ANFIS hybridized with Grey Wolf Optimizer, Constr. Build. Mater. 232 (2020) 117266. https://doi.org/10.1016/j.conbuildmat.2019.117266.

[76] S.E.F. and C. Lebiere, The Cascade-Correlation Learning Architecture, 1992 SEG Annu. Meet. (1992) 1136–1139. https://doi.org/10.1190/1.1821929.

[77] J.A. Anderson, Cognitive and Psychological Computation with Neural Models, IEEE Trans. Syst. Man Cybern. SMC-13 (1983) 799–815. https://doi.org/10.1109/TSMC.1983.6313074.

[78] G.E. Dahl, T.N. Sainath, G.E. Hinton, Improving deep neural networks for LVCSR using rectified linear units and dropout, ICASSP, IEEE Int. Conf. Acoust. Speech Signal Process. - Proc. (2013) 8609–8613. https://doi.org/10.1109/ICASSP.2013.6639346.

[79] A.F. Agarap, Deep Learning using Rectified Linear Units (ReLU), (2018) 2–8. http://arxiv.org/abs/1803.08375.

[80] W. Hong, Wavelet Gradient Boosting Regression Method Study in Short-Term Load Forecasting, Smart Grid. (2015) 189–196.

[81] J. Brownlee, A Gentle Introduction to the Gradient Boosting Algorithm for Machine Learning, (2016). https://machinelearningmastery.com/gentle-introduction-gradient-boosting-algorithm-machine-learning/ (accessed July 7, 2022).

[82] T. Head, M. Kumar, H. Nahrstaedt, G. Louppe, I. Shcherbatyi, scikit-optimize/scikit-optimize: v0.9.0 (Version v0.9.0), (2021). https://doi.org/10.5281/zenodo.5565057 (accessed July 7, 2022).

[83] T. Chen, C. Guestrin, XGBoost: A Scalable Tree Boosting System Tianqi, in: Proc. 22nd ACM SIGKDD Int. Conf. Knowl. Discov. Data Min., ACM, New York, NY, USA, 2016: pp. 785–794. https://doi.org/10.1145/2939672.2939785.

[84] M.S. Alajmi, A.M. Almeshal, Predicting the tool wear of a drilling process using novel machine learning XGBoost-SDA, Materials (Basel). 13 (2020) 1–16. https://doi.org/10.3390/ma13214952.

[85] R. Santhanam, N. Uzir, S. Raman, S. Banerjee, Experimenting XGBoost Algorithm for Prediction and Classification of Different Ramraj S , Nishant Uzir , Sunil R and Shatadeep Banerjee Experimenting XGBoost Algorithm for Prediction and Classi fi cation of Different Datasets, Int. J. Control Theory Appl. 9 (2017) 651–662.

[86] T. Hastie, R. Tibshirani, J. Friedman, The Elements of Stastical Learning: Data Mining, Inference and Prediction, Second, Springer, 2008.

[87] R. Pavan, M. Nara, S. Gopinath, N. Patil, Bayesian optimization and gradient boosting to detect phishing websites, 2021 55th Annu. Conf. Inf. Sci. Syst. CISS 2021. (2021) 1–5. https://doi.org/10.1109/CISS50987.2021.9400317.

[88] N. Arkalgud, Logistic Regression for Spam Filtering, (2016) 1–8.

[89] A.V. Dorogush, V. Ershov, A. Gulin, CatBoost: gradient boosting with categorical features support, (2018) 1–7. http://arxiv.org/abs/1810.11363.

[90] M.I. Sameen, B. Pradhan, S. Lee, Self-Learning Random Forests Model for Mapping Groundwater Yield in Data-Scarce Areas, Nat. Resour. Res. 28 (2019) 757–775. https://doi.org/10.1007/s11053-018-9416-1.

[91] C. Thornton, F. Hutter, H.H. Hoos, K. Leyton-Brown, Auto-WEKA: Combined selection and hyperparameter optimization of classification algorithms, Proc. ACM SIGKDD Int. Conf. Knowl. Discov. Data Min. Part F1288 (2013) 847–855. https://doi.org/10.1145/2487575.2487629.

[92] F. Hutter, H. Hoos, K. Leyton-Brown, Sequential Model-Based Optimization for General Algorithm Configuration Lecture Notes in Computer Science, Int. Conf. Learn. Intell. Optim. (2011) 507–523. https://www.cs.ubc.ca/~hutter/papers/10-TR-SMAC.pdf.

[93] G. Rong, S. Alu, K. Li, Y. Su, J. Zhang, Y. Zhang, T. Li, Rainfall Induced Landslide Susceptibility Mapping Based on Bayesian Optimized Random Forest and Gradient Boosting Decision Tree Models—A Case Study of Shuicheng County, China, Water. 12 (2020) 3066. https://doi.org/10.3390/w12113066.

[94] W. Zhang, C. Wu, H. Zhong, Y. Li, L. Wang, Prediction of undrained shear strength using extreme gradient boosting and random forest based on Bayesian optimization, Geosci. Front. 12 (2021) 469–477. https://doi.org/10.1016/j.gsf.2020.03.007.

[95] J. Bergstra, B. Komer, C. Eliasmith, D. Warde-Farley, Preliminary evaluation of hyperopt algorithms on HPOLib, ICML Work. AutoML. (2014) 2013.

[96] J. Bergstra, R. Bardenet, Y. Bengio, B. Kégl, Algorithms for hyper-parameter optimization, Adv. Neural Inf. Process. Syst. 24 25th Annu. Conf. Neural Inf. Process. Syst. 2011, NIPS 2011. (2011) 1–9.

[97] Y. Freund, R.E. Schapire, A Decision-Theoretic Generalization of On-Line Learning and an Application to Boosting, J. Comput. Syst. Sci. 55 (1997) 119–139. https://doi.org/10.1006/jcss.1997.1504.

[98] O. Sagi, L. Rokach, Ensemble learning: A survey, Wiley Interdiscip. Rev. Data Min. Knowl. Discov. 8 (2018) 1–18. https://doi.org/10.1002/widm.1249.
